# Fresh perspectives on an established technique: Pulsed amplitude modulation chlorophyll *a* fluorescence

**DOI:** 10.1002/pei3.10073

**Published:** 2022-03-31

**Authors:** Guanqiang Zuo, Robert M. Aiken, Naijie Feng, Dianfeng Zheng, Haidong Zhao, Thomas J. Avenson, Xiaomao Lin

**Affiliations:** ^1^ Department of Agronomy Kansas State University Manhattan Kansas USA; ^2^ Northwest Research‐Extension Center Kansas State University Colby Kansas USA; ^3^ College of Coastal Agricultural Science Guangdong Ocean University Zhanjiang China; ^4^ Shenzhen Research Institute of Guangdong Ocean University Shenzhen China; ^5^ Department of Plant Sciences University of Cambridge Cambridge UK

**Keywords:** chlorophyll *a* fluorescence, light sources, measurement, photosynthesis, plant stress

## Abstract

Pulsed amplitude modulation (PAM) chlorophyll *a* fluorescence provides information about photosynthetic energy transduction. When reliably measured, chlorophyll *a* fluorescence provides detailed information about critical in vivo photosynthetic processes. Such information has recently provided novel and critical insights into how the yield potential of crops can be improved and it is being used to understand remotely sensed fluorescence, which is termed solar‐induced fluorescence and will be solely measured by a satellite scheduled to be launched this year. While PAM chlorophyll *a* fluorometers measure fluorescence intensity *per se*, herein we articulate the axiomatic criteria by which instrumentally detected intensities can be assumed to assess *fluorescence yield*, a phenomenon quite different than fluorescence intensity and one that provides critical insight about how solar energy is variably partitioned into the biosphere. An integrated mathematical, phenomenological, and practical discussion of many useful chlorophyll *a* fluorescence parameters is presented. We draw attention to, and provide examples of, potential uncertainties that can result from incorrect methodological practices and potentially problematic instrumental design features. Fundamentals of fluorescence measurements are discussed, including the major assumptions underlying the signals and the methodological caveats about taking measurements during both dark‐ and light‐adapted conditions. Key fluorescence parameters are discussed in the context of recent applications under environmental stress. Nuanced information that can be gleaned from intra‐comparisons of fluorescence‐derived parameters and intercomparisons of fluorescence‐derived parameters with those based on other techniques is elucidated.

## Graphical abstract

PAM chlorophyll a fluorometers detect ‘intensities’ that are presumed to represent chlorophyll fluorescence yield (ΦF), the latter of which provides information about how photosynthetic organisms variably partition solar energy into the biosphere. Yet exactly how and why detected intensities can be assumed to represent ΦF is not always explicitly stated in the literature. Therefore, from the outset, we derive the meaning of ΦF from first principles and state what criteria are required for instrumentally detected intensities to represent ΦF. We then include a section where the mathematical expressions of key ΦF parameters are juxtaposed with their respective experimental phenomena.

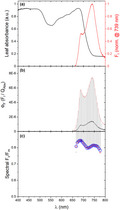

## 1 INTRODUCTION

The world is facing significant environmental challenges on a variety of scales, some of which threaten survival on fundamental levels. Food security, for example, could very likely remain a worldwide concern for several decades and beyond due to a growing global population, and it will likely be further challenged by future climate change (Change, 2020; Tallis et al., 2018). A doubling of crop production is projected to be needed by 2050 (Ray et al., 2013), and such demands require a doubling of productivity per hectare due to limited arable land (Ort et al., 2015). The decades of research that have significantly advanced our knowledge of “leaf‐level” photosynthesis may very well enable the conception of novel hypotheses that ultimately play a critical role in meeting these future challenges (Kromdijk et al., 2016; Zhu et al., 2010).

**FIGURE 1:**
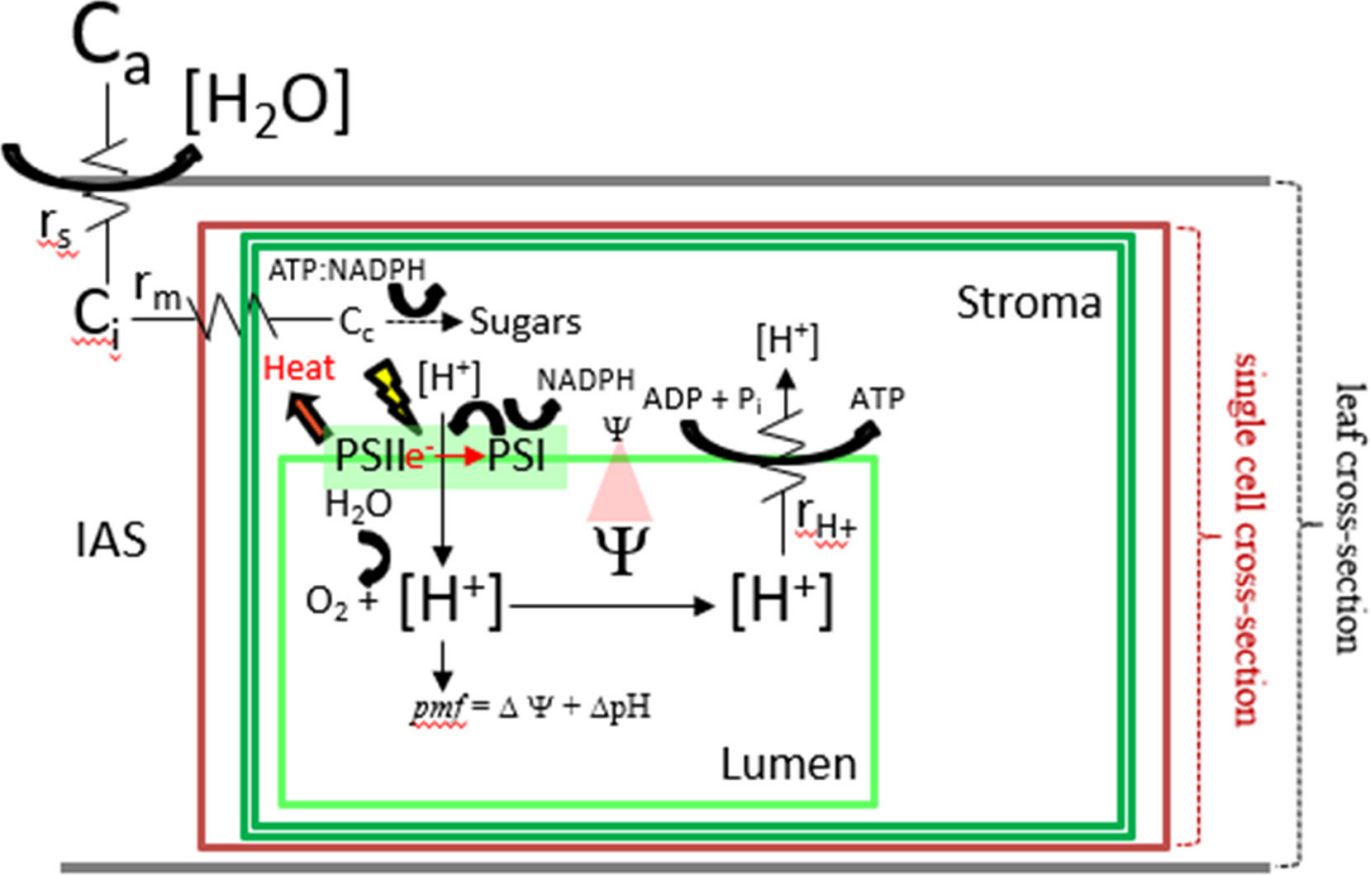
The adaxial (upper) and abaxial (lower) boundaries of a hypothetical leaf cross‐section are represented as horizontal lines (gray). The intercellular air space (IAS) is indicated. The hypothetically “merged” cell wall and plasma membrane of a single cell are represented as larger of the rectangles (brown) within the leaf cross‐section. The double‐membrane chloroplast (dark green) fills most of the hypothetical volume of the cell. A single thylakoid membrane is represented as the smallest rectangle (light green) within the chloroplast. Atmospheric (*C*_a_), inter‐cellular air space (*C*_i_), and chloroplastic (*C*_c_) [CO_2_] font sizes depict relative differences in the respective concentrations. Differences in the respective CO_2_ concentrations are due to stomatal (*r*_s_) and mesophyll (*r*_m_) resistances. The *r*_s_, *r*_m_, and ATP synthase ($r_{H}^{+}$) resistances are indicated by electronic resistor symbols; the reciprocals of each of the resistances equal the corresponding conductances (*g*_s_, *g*_m_, and $g_{H}^{+}$). Photosystem (PS) II and PSI are intra‐thylakoid membrane, multi‐subunit complexes that bind an assortment of light‐harvesting pigments. Light‐driven (yellow lightning bolt), proton‐coupled linear electron flux (LEF) is initiated at PSII, with H_2_O (lumen side of thylakoid membrane) serving as the source of electrons (e^−^), whereas NADPH (stromal compartment of chloroplast) is the predominant “sink” for the electrons. LEF and cyclic electron flux (CEF) around PSI establish a consequent proton gradient, or proton motive force (*pmf*), across the thylakoid membrane. Because protons are charged, the *pmf* is comprised of trans‐thylakoid membrane pH (ΔpH) and electric field (ΔΨ) gradients, the latter being represented by the red, transparent triangle between Ψ‐symbols on opposite sides of the thylakoid membrane. Font sizes of proton symbols (H^+^) indicate relative differences in proton concentrations (brackets). Heat dissipation away from PSII (red arrow) is indicative of energy‐dependent quenching (*q*_E_) of excess absorbed energy. As protons move down their electrochemical gradient from the thylakoid lumen and into the chloroplast stroma through the ATP synthase, ATP is generated. The ATP and NADPH are used to assimilate *C*_c_ into sugars and power other metabolic processes not indicated

Leaf‐level photosynthesis is highly and delicately coordinated. As a generic description of leaf‐level photosynthesis, which does not take into consideration the nuances of all “types” of photosynthesis (e.g., C_3_, C_4_, and CAM, etc.), Figure 1 depicts the idea that atmospheric CO_2_ (*C*_a_) must diffuse through stomatal apertures into the intercellular air space within a leaf. Stomatal pores impose a variable amount of resistance to CO_2_ diffusion (*r*_s_; the reciprocal of r_s_ is stomatal conductance, *g*_s_; Assmann, 1999). The intercellular CO_2_ (*C*_i_) must then diffuse through the mesophyll pathway, which includes the cell wall, the plasma membrane, the cytosol, the double‐membrane of the chloroplast, and the chloroplast stroma, the latter being the site of carboxylation of chloroplastic CO_2_ (*C*_c_; Farquhar et al., 1980). The mesophyll pathway is tortuous and contributes to the resistance of CO_2_ diffusion (*r*_m_), the reciprocal of which is termed mesophyll conductance (or *g*_m_), to the site of carboxylation (Pons et al., 2009). Carboxylation involves the covalent incorporation of atmospheric CO_2_ into the five‐carbon molecule ribulose‐1,5‐bisphosphate (RUBP) and is an anabolic process that will be referred to herein as net CO_2_ assimilation (*A*_Net_; Long & Bernacchi, 2003). *A*_Net_ ultimately requires regeneration of RUBP, consequently rendering it dependent on rigidly stoichiometric amounts of chemical energy, namely, ATP and NADPH (Walker et al., 2020). The chemical energy is produced by delicately coordinated conversion of light energy within thylakoid membranes and involves proton‐coupled “linear” and “cyclic” electron fluxes (LEF and CEF, respectively; Cruz & Avenson, 2021; Cruz et al., 2005). On the one hand, LEF and its consequent production of NADPH and generation of a trans‐thylakoid proton motive force (*pmf*), is mediated by both photosystem (PS) II and PSI, each of which binds an assortment of pigments, but predominantly chlorophylls *a* and *b* (Merchant & Sawaya, 2005). On the other hand, CEF is solely associated with PSI and functions to augment the *pmf* (Livingston et al., 2010; Walker et al., 2014), putatively playing roles in balancing the ATP:NADPH output ratio and regulation of light capture (Livingston et al., 2010; Walker et al., 2014). Total *pmf* consists of gradients of both pH (ΔpH) and electric field (ΔΨ; Cruz et al., 2001) and it drives the synthesis of ATP as protons (H^+^) move “down” their electrochemical gradient from the thylakoid lumen and into the chloroplast stroma through the chloroplast ATP synthase (Capaldi &Aggeler, 2002), an enzyme which imposes variable resistance to proton efflux ($r_{H}^{+}$; the reciprocal of $r_{H}^{+}$ is termed the proton conductance, $g_{H}^{+}$; Avenson et al., 2005; Kanazawa & Kramer, 2002). Except under relatively low light intensities, and even during otherwise environmentally favorable conditions, light energy can be routinely absorbed faster than the resultant chemical energy can be consumed (Walker et al., 2020). Such circumstances have the potential to be catastrophic due to the increased probability of side reactions that give rise to the formation of deleterious forms of reactive oxygen species (ROS), including singlet oxygen (^1^O_2_) and the superoxide radical (O_2_^−^; Niyogi, 1999). Non‐photochemical quenching (NPQ), a composite of PSII‐associated, photoprotective processes (Müller et al., 2001), functions to benignly dissipate excess absorbed light energy, thereby minimizing the potential for photooxidative damage (Niyogi, 1999). The predominant component of NPQ harmlessly dissipates excess energy as heat and is referred to as energy‐dependent quenching of excitons (*q*_E_; Crofts & Yerkes, 1994), the rapid reversibility of which, as during the transient shading of a leaf during the passing of a cloud or wind‐driven shading by the chaotic movements of leaves within canopies, is partially dependent on processes that reversibly modulate the magnitude of the ΔpH component of the *pmf* (Bennett et al., 2019; Kramer et al., 2003).

Optimizing various aspects of these coordinated processes of leaf‐level photosynthesis may be the only means of improving yield potential (*Y*_P_), which is the optimal grain yield that a crop can achieve (Zhu et al., 2010). *Y*_P_ is defined with respect to ideal resource management, which includes nutrients, water, and the absence of both biotic and abiotic stress factors (Slattery et al., 2017; Zhu et al., 2010):

(1) $$Y_{P}=0.487*S_{t}*\varepsilon_{i}*\varepsilon_{c}*\varepsilon_{p},$$

where *S*_t_ represents the total incident solar radiation across the whole growing season; 0.487 is an approximation of the proportion of photosynthetically active radiation (PAR) in *S*_t_; *ε*_i_, *ε*_c_, and *ε*_p_ correspond to the efficiencies of radiation interception, the conversion of intercepted radiation into biomass, and partitioning of biomass into a harvestable product (harvest index, HI), respectively. Therefore, any improvements in *ε*_i_, *ε*_c_, and/or *ε*_p_ could potentially enhance *Y*_P_. However, studies have shown, as a result of agronomic practices (Zhu et al., 2010), that *ε*_i_ and *ε*_p_ for many current crops are close to their theoretical maxima, leaving improvements in *ε*_c_, which is directly related to leaf‐level photosynthesis, as a promising, and perhaps only, process by which *Y*_p_ is capable of being improved, either through transgenics or traditional breeding (Zhu et al., 2010).

Many crops are grown under conditions that are anything but ideal, and are rather grown under unavoidable extremes of various environmental stresses, paramount of which is drought, an environmentally, and often intermittent, stressful condition widely acknowledged to be the primary limitation to worldwide food productivity (Boyer, 1982). Leaf‐level photosynthesis is highly flexible in response to such environmental fluctuations, yet there is significant interest in improving yields under such conditions by identifying, understanding, and transgenically, or through traditional breeding, improving leaf‐level photosynthetic processes. In C_3_ leaves, it is well known that a fundamental progression of leaf‐level drought responses involves decreased g_s_, but such decreases have also been shown to be accompanied by, depending upon the drought severity and species, decreased g_m_ (Flexas et al., 2012). The decrease in *g*_s_ functions is to conserve intra‐plant water content, whereas the rationale for the decrease in *g*_m_ is currently being intensely investigated because of its potential to improve yield under drought conditions (Flexas et al., 2008). Because of diminished *g*_s_ and *g*_m_, the concentration of *C*_c_ is driven down, consequently lowering both A_Net_ and LEF (Lal & Edwards, 1996). Since *q*_E_ is partially dependent upon the ΔpH component of the *pmf* (Crofts & Yerkes, 1994), and LEF is the predominant proton‐coupled process by which the ΔpH of the thylakoid lumen is regulated, such a combination of drought‐induced changes predicts catastrophic failure (Kanazawa & Kramer, 2002). Diminished LEF under such conditions, and in the absence of compensatory adjustments, would be expected to result in a decrease in the magnitude of the ΔpH and consequently *q*_E_ (Kanazawa & Kramer, 2002). Given that light intensities can remain constant under drought, such a combination of circumstances would be predicted to deleteriously enhance the absorption of excess light energy and thereby exacerbate photooxidative stress (Kanazawa & Kramer, 2002). Knowledge of these complexities, and their regulation, is critical to devising crop yield improvement strategies.

Understanding the complexities of the coordinated processes of leaf‐level photosynthesis over the wide range of environmental conditions experienced by plants in nature will likely require combinatorial measurement techniques and methodologies (Cruz & Avenson, 2021), none of which currently exist within a single commercially available instrument. This circumstance belies the fact that it is an understanding of these coordinated details (Morales et al., 2018), including, for example, the inter‐relationships between *A*_Net_, *g*_m_, and $g_{H}^{+}$, measured as near simultaneously as technically possible, that will likely play a role in successfully addressing the abovementioned challenges. Nevertheless, there are several commercially available instruments that can measure partial aspects of leaf‐level photosynthesis. Absorption of infrared light by H_2_O and CO_2_ at distinct wavelengths enabled the development of instrumentation for measuring gas exchange parameters like A_Net_, g_s_, and several other direct and indirect gas‐exchange parameters (Farquhar et al., 1980; Long & Bernacchi, 2003; Von Caemmerer, & v.,, & Farquhar, G. D., 1981). A technique based on differential absorption of visible light, which is growing in interest, is termed Dark Interval Relaxation Kinetic (DIRK) analyses of the electrochromic shift (ECS; Sacksteder & Kramer, 2000). The ECS is a trans‐thylakoid (Figure 1), ΔΨ‐induced shift in the absorption spectra of certain carotenoids, the maximum amplitude of which occurs at ~520 nm, that are bound by intra‐thylakoid‐membrane light‐absorbing proteins (Witt & Zickler, 1973). DIRK analyses of the ECS have been extensively adapted to monitor leaf‐level photosynthetic processes that cause changes in the trans‐thylakoid ΔΨ (Cruz et al., 2001; Kramer, Avenson, & Edwards, 2004; Sacksteder et al., 2000). LEF is a proton‐coupled electron transfer process and DIRK analyses of the ECS have proven capable of providing explicit details about various aspects of the resultant “proton circuit” of photosynthesis (Avenson et al., 2005), including estimates of $g_{H}^{+}$, the magnitude of the light‐induced *pmf*, and the relative partitioning of *pmf* into ΔΨ and ΔpH (Avenson et al., 2004; Cruz et al., 2001; Davis et al., 2016; Kanazawa & Kramer, 2002). In addition, various techniques based on measurements of chlorophyll *a* fluorescence have been developed and provide diverse information about many detailed aspects of the energy‐producing reactions of photosynthesis, including estimates of LEF, NPQ, and the relative parsing of NPQ into its constituent components (Müller et al., 2001).

Herein we review an established technique for measuring a variety of leaf‐level processes using chlorophyll *a* fluorescence, namely, pulsed amplitude modulation (PAM) chlorophyll *a* fluorescence, but we do so with the aim of comprehensively linking axiomatic principles underlying the mathematics, methodologies, instrumentation, phenomenology, and the plethora of physiologic information that can be learned from intra‐ and intercomparisons of relevant parameters. We do so with the hope of encouraging users, especially those who are inexperienced and want to contribute to, and be involved in, exciting developments within the scope of their research. We think this is relevant given the technique's recent role in formulating and testing novel hypotheses concerning ways of improving *Y*_P_ (Kromdijk et al., 2016), as well as its role in assessing unique drought‐related processes (Kanazawa & Kramer, 2002). Moreover, PAM chlorophyll *a* fluorescence is also playing a role in developing and understanding remotely‐sensed fluorescence, or solar‐induced fluorescence (SIF; Berry, 2018), thereby providing a link between leaf‐level and remotely sensed (i.e., satellite‐ and tower‐based) measurements of chlorophyll *a* fluorescence (Magney et al., 2017; Magney et al., 2019). PAM chlorophyll *a* fluorescence measures a useful phenomenon termed “fluorescence yield” (Φ_F_), the meaning of which can seem elusive, so we explicitly define it by mathematically deriving and describing its axiomatic meaning from first principles. To promote a more intuitive understanding of the various Φ_F_ parameters that are obtained from PAM chlorophyll *a* fluorescence, an entire section is devoted to describing their mathematical definitions in conjunction with a plot of the corresponding phenomenology, which involves changes in Φ_F_ in response to various experimental manipulations. This section also includes a thorough explanation of the underlying assumptions, as well as user, instrument, and methodological caveats that underly accurate measurement of the various Φ_F_ parameters. Data are presented to illustrate errors associated with improper experimental methodology, and experiments are described and proposed to ensure that measurements of the various Φ_F_ parameters are properly made. Lastly, a thorough description of several meaningful physiologic processes that can be derived from the various Φ_F_ parameters will be presented, as well as unique information that can be obtained through intra‐comparisons, namely, Φ_F_‐derived physiologic processes with one another, and intercomparisons, which means comparing Φ_F_‐derived physiologic processes with those based on other techniques.

## 2 FLUORESCENCE INTENSITY VERSUS YIELD: THE AXIOMATIC BASIS FOR INFERRING DIFFERENTIAL PARTITIONING OF SOLAR ENERGY

The concept of Φ_F_ is useful for assessing how light energy absorbed by chlorophyll, the predominant light‐absorbing pigment on the planet, is variably partitioned into the biosphere by plants and other photosynthetic organisms. Intrinsic concepts underlying various mathematical formulations of Φ_F_, by which the fluorescence signals are measured using different techniques, begin with considering illumination or absorption of photons (h*ν*) in a population of ground‐state chlorophyll (Chl^gs^) molecules in solution. This system will generate a population of *singlet* excited chlorophyll molecules (Chl^1*^) described by (Krause & Weis, 1991):

(2) $${\mathrm{Chl}}^{\mathrm{gs}}+h\nu \to {\mathrm{Chl}}^{1*}.$$

From the steady‐state, cessation of illumination will abruptly initiate decay of Chl^1*^ over time, a process that can be described as a first‐order system (Nobel, 1999):

(3) $$\frac{d\left[{\mathrm{Chl}}^{1*}\right]}{\mathrm{dt}}=-k_{{\mathrm{Chl}}^{1*}}\left[{\mathrm{Chl}}^{1*}\right],$$

where *k*_Chl1*_ corresponds to the overall *rate* constant by which Chl^1*^ can return to the ground state. A critical expression for the following

discussion involves *k*_Chl1*_ and is defined as:

(4) $$\frac{1}{\tau_{{\mathrm{Chl}}^{1*}}}=k_{{\mathrm{Chl}}^{1*}}=\sum_{j}k_{j}=\sum_{j}\frac{1}{\tau_{j}},$$

where $\tau_{{\mathrm{Chl}}^{1*}}$ corresponds to the overall *time* constant for decay of Chl^1*^; $\sum_{j}k_{j}$ and $\sum_{j}\frac{1}{\tau_{j}}$ correspond to the sums of *j*th rate and reciprocal time constants, respectively, for intrinsic mechanisms by which Chl^1*^ can decay to the ground state. Based on Equation (4), $\tau_{{\mathrm{Chl}}^{1*}}$ and $k_{{\mathrm{Chl}}^{1*}}$ equate to $\frac{1}{\sum_{j}\frac{1}{\tau_{j}}}$ and $\sum_{j}k_{j}$, respectively. Given these definitions, and several derivative steps (not shown), Equation (3) can be solved as a single exponential decay function (Nobel, 1999):

(5) $$\left[{\mathrm{Chl}}^{1*}\right]\left(t\right)=\left[{\mathrm{Chl}}^{1*}\right]\left(0\right)e^{-k_{{\mathrm{Chl}}^{1*}}*t},$$

where $\left[{\mathrm{Chl}}^{1*}\right]\left(t\right)$ and $\left[{\mathrm{Chl}}^{1*}\right]\left(0\right)$ correspond to the concentrations of singlet excited chlorophyll at time “*t*” and time *t* = 0, respectively, the latter representing the initial Chl^1*^ upon cessation of illumination. An especially useful expression linking rate and time constants can be obtained from Equation (5) by assuming that “*t*” corresponds to $\tau_{{\mathrm{Chl}}^{1*}}$:

(6) $$\left[{\mathrm{Chl}}^{1*}\right]\left(\tau_{{\text{Chl}}^{1*}}\right)=\left[{\mathrm{Chl}}^{1*}\right]\left(0\right)e^{-k_{{\text{Chl}}^{1*}}{\tau_{{\text{Chl}}^{1*}}}_{}}.$$

Based on the abovementioned definitions of $\tau_{{\mathrm{Chl}}^{1*}}$ and $k_{{\mathrm{Chl}}^{1*}}$, Equation (6) simplifies to an expression that defines the essence of the meaning of $\tau_{{\mathrm{Chl}}^{1*}}$:

(7) $$\left[{\mathrm{Chl}}^{1*}\right]\left(\tau_{{\text{Chl}}^{1*}}\right)=\left[{\mathrm{Chl}}^{1*}\right]\left(0\right)e^{-1}.$$

Thus, by convention, $\tau_{{\mathrm{Chl}}^{1*}}$ is defined as the time it takes for ${\left[\mathrm{Chl}\right]}^{1*}\;\left(0\right)$ to decay (decrease) to 1/e, for example, to 37%. An implication of Equations (6) and (7) is that:

(8) $$\left(\sum_{j}k_{j}\right)\tau_{{\mathrm{Chl}}^{1*}}=1.$$

Therefore, if, for example, the *j*th rate constant for any mechanism were to be experimentally manipulated to change, a fundamental tenant of the PAM chlorophyll *a* fluorescence technique (Schreiber, 2004), then $\tau_{{\mathrm{Chl}}^{1*}}$ must necessarily change in an inverse manner.

The inverse interplay between rate and time constants provides a link to different conceptual formulations of *yield* (Φ). A *descriptive* manner of defining Φ_F_ is that it corresponds to the proportion of Chl^1*^ that decays by, or dissipates absorbed solar energy as, fluorescence relative to the proportion of Chl^1*^ that decays, or is converted to other forms of energy, by the sum of all dissipative mechanisms. In solution, for example, Chl^1*^ can decay via fluorescence (F), internal conversion (IC), and intersystem crossing (ISC) into the triplet state (Bowers & Porter, 1967). The mechanism that dissipates chlorophyll excited states the fastest, or exhibits the greatest *k* or shortest *τ*, will be the highest “yielding.” The yield associated with any given mechanism can thus be conceptually described according to either rate or time constants. Based on solution‐dependent mechanisms (above), and the abovementioned definitions, Φ_F_ can be conceptually defined according to time and rate constants as (Nobel, 1999):

(9) $$\Phi_{F}=\frac{\frac{1}{\sum \left(\frac{1}{\tau_{F}}+\frac{1}{\tau_{\mathrm{IC}}}+\frac{1}{\tau_{\mathrm{ISC}}}\right)}}{\tau_{F}}=\frac{k_{F}}{\sum \left(k_{F}+k_{\mathrm{IC}}+k_{\mathrm{ISC}}\right)}.$$

While expression of Φ_F_'s according to rate constants (*k*_*j*_) is the convention predominantly found in the literature describing PAM chlorophyll *a* fluorescence measurement of leaf‐level phenomena (Kramer, Johnson, et al., 2004; Papageorgiou, 2007), alternative expressions involving time constants, which are predicated on measured fluorescence lifetimes, can be obtained that are based on alternative techniques (Bennett et al., 2019; Park et al., 2017).

It should be noted that PAM chlorophyll *a* fluorometers do not measure Φ_F_ *per se*. Rather a useful, but by no means sole, expression for describing Φ_F_ that is based on instrumentally measurable quantities represents chlorophyll *a* fluorescence emission per unit absorbed light (slightly modified from Krause & Weis, 1991):

(10) $$\Phi_{F}=\frac{{\mathrm{dF}}_{I}}{{\mathrm{dQ}}_{\mathrm{Abs}}},$$

where *dF*_I_ and *dQ*_Abs_ correspond to fluorescence (μmol m^−2^ s^−1^) and absorbed light intensities (μmol m^−2^ s^−1^), respectively. Therefore, instrumentally “detected” intensities can provide quantitative information about Φ_F_ if: (1) they explicitly represent changes in F_I_'s elicited by a known change in *Q*_Abs_; or (2) they explicitly represent changes in *F*_I_'s elicited by a *constant Q*_Abs_. PAM chlorophyll *a* fluorescence is predicated on the latter. Briefly, the modulating pulse electronic componentry is designed such that the amplitudes of each light pulse are servo‐controlled (i.e., to ensure constant amplitudes of every light pulse, obviating the need to explicitly know *Q*_Abs_), the pulses typically have “rise” and “fall” time constants on the 100's of nanosecond timescale, and they are “ON” for a duration of ~1 μs at frequencies no greater (i.e., depending upon experimental circumstances) than 250 kHz (i.e., 4 μs between pulses). The detector componentry consists of optical filters that ensure detection of near‐infrared light (i.e., fluorescence), and the detector electronics are “synced” with those of the modulated light so as to “integrate” detected *F*_I_'s for a duration of 1 μs. In short, the modulated light illumination and detection electronics are exquisitely designed so as to illuminate the sample of interest with light pulses of *constant intensity* and explicitly detect *F*_I_'s associated with said pulses (see below for experimental manipulations involving modulated light).

Plants and other photosynthetic organisms possess additional mechanisms by which absorbed solar energy can be dissipated. When considering Φ_F_ measured from photosynthetic organisms that possess such additional physiologic mechanisms that compete with F, IC, and ISC for dissipating light energy absorbed by chlorophyll, namely, PSII‐mediated photochemistry (PC), which initiates LEF, and NPQ, the first‐order system should also include *apparent* rate constants for PC and NPQ (*K*_*P*C_ and *K*_NPQ_, respectively):

(11) $$\Phi_{F}=\frac{k_{F}}{\sum \left(k_{F}+k_{\mathrm{IC}}+k_{\mathrm{ISC}}+k_{\mathrm{PC}}*Q_{A}+K_{\mathrm{NPQ}}\right)}.$$

The “apparent” nature of these rate constants is meant to convey the notion that an *intrinsic* rate constant (“*k*”; lowercase) is coupled to some other essential component that impacts the magnitude of the apparent rate constant. *K*_PC_, for example, can be understood as the product (see Equation 11) of an intrinsic rate constant (*k*_PC_) for photochemical electron transfer mediated by redox species within the PSII reaction center (Lavergne & Trissl, 1995) and the *neutral* state of the primary electron acceptor, namely, a bound quinone species (*Q*_A_) within the PSII reaction center. It is widely accepted that the redox state of *Q*_A_ can be experimentally manipulated to modulate the emanation of chlorophyll *a* fluorescence from the light‐absorbing pigment “antenna” of PSII (Schatz et al., 1988). In the equations discussed herein, ‘*Q*_A_’ will be assumed to represent the ensemble, or total, *proportion* of the oxidized, or often termed the “open” (Papageorgiou, 2007), state of *Q*_A_. The apparent rate constant for NPQ can also be conceptually parsed into the sum of intrinsic rate constants for at least (Malnoë, 2018) three distinct phenomena:

(12) $$K_{\mathrm{NPQ}}=\sum \left(k_{\mathrm{qE}}\left[\mathrm{Zea}\right]\left[P\right]\mathrm{\Delta pH}+k_{\mathrm{qT}}+k_{\mathrm{qI}}\right),$$

where *k*_qE_, *k*_qT_, and *k*_qI_ correspond to intrinsic rate constants for *q*_E_, state transitions (*q*_T_), for example, a process by which pigment‐binding proteins reversibly associate with, and thereby balance delivery of absorbed light energy to, PSII and PSI as necessary (Depège et al., 2003), and a somewhat ambiguous mechanism putatively involving inhibition of PSII (*q*_I_; Malnoë, 2018), respectively. q_E_ is typically the predominant component of NPQ (Müller et al., 2001), and its rapidly reversible nature has recently attracted significant attention because of its potential to be genetically manipulated for the purpose of improving crop yield (Kromdijk et al., 2016). The carotenoid zeaxanthin (Zea) and the PSII‐associated protein PsbS (P) are necessary and sufficient for steady‐state q_E_, but they do not explicitly account for the dynamic reversibility of *q*_E_ that is putatively necessary for plant survival in nature (Külheim et al., 2002). While q_E_ has been shown to be rapidly reversible in response to short (seconds‐to‐minutes), repetitive light‐to‐dark fluctuations, simultaneous measurements of Zea were nonetheless shown to remain constant, indicating that changes in Zea *per se* were not responsible for the changes in *q*_E_. Modeling suggested that the rapid changes in *q*_E_ were due to concomitantly rapid fluctuations in ΔpH (Park et al., 2017). Given that fluorescence measurements have been used in elucidating the functionality of indispensable processes like *q*_E_, measurements of “photosynthetic” Φ_F_ have been indispensable in obtaining quantitative information about how absorbed solar energy is variably partitioned, or *converted*, into forms of energy in the biosphere.

## 3 PAM CHLOROPHYLL *A* FLUORESCENCE: THE ESSENCE OF A CLEVER TECHNIQUE

**FIGURE 2:**
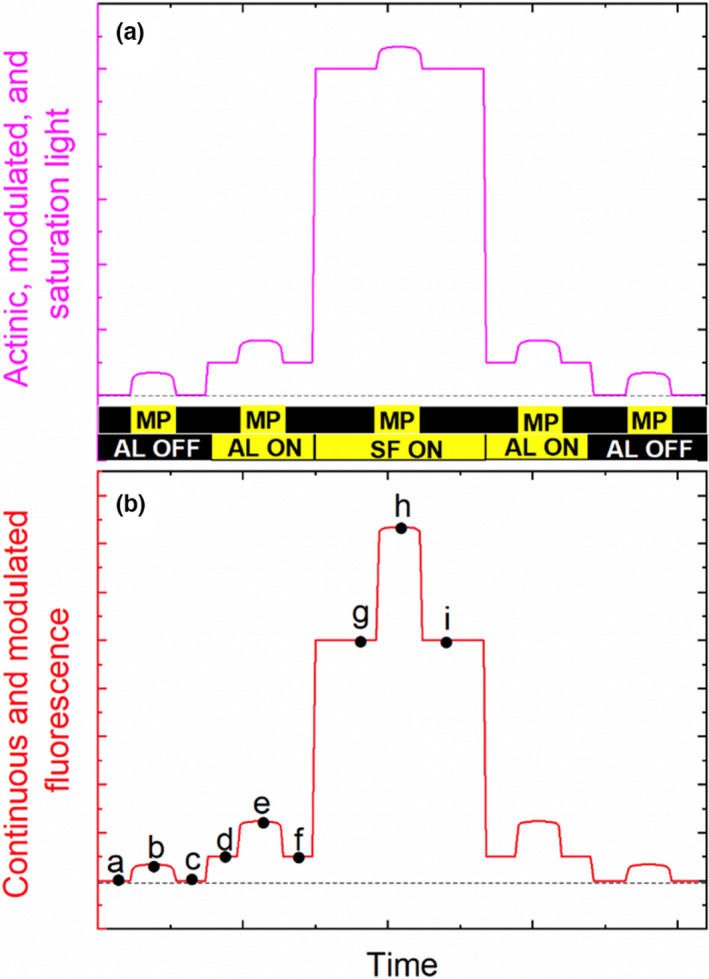
Schematic depiction of light sources used during experiments (a) cumulative light intensities that are produced by the modulated light (ML), actinic light (AL), and saturation flash (SF) light sources are schematically depicted. Individual modulated pulses (MPs) are shown at the bottom of the figure. The bar at the very bottom represents continuous, non‐modulated light sources, AL and SF light; (**b**) the cumulative fluorescence signals elicited by the respective light sources are schematically depicted; modulated fluorescence (MF) and continuous fluorescence (CF) are elicited by MPs and AL and SF, respectively. The letters represent time points at which the ML and detector electronics facilitate the detection of fluorescence intensities. Demodulation of the fluorescence intensities into MF and CF is described in the main text

An evolution in chlorophyll *a* fluorescence measurement occurred with the development of PAM chlorophyll *a* fluorometry, which brought about a “renaissance” of interest in plant research (Schreiber, 1986). Over the decades, several commercially available PAM chlorophyll *a* fluorometers have been integrated into photosynthesis *systems* that also incorporate such techniques involving Infrared Gas Analysis, for example, gas exchange, and are thus equipped with multiple light sources having different functionalities. Figure 2a shows a highly schematic depiction of the various light sources with which such photosynthesis systems can be equipped. The three illumination sources depicted are the: (1) modulation light (ML); (2) actinic light (AL); and (3) saturation flash (SF) light, the latter of which is typically several fold higher than full sunlight, but only for a short duration of time, typically ~1 s (Schreiber, 2004). An illumination source that is not shown, but that will be discussed herein, is a far‐red (FR) light source. The ML source consists of a continual train of light “pulses,” or modulated pulses (MPs), that is always ON during an experiment and is typically, although not exclusively, associated with an independent array of light‐emitting diodes (LEDs). The *integrated* intensity (*I*_I_) of the ML, a critical parameter to be aware of (see section below), is a function of intrinsic pulse characteristics:

(13) $$I_{I}=p_{I}*p_{F}*p_{D},$$

where *p*_I_, *p*_F_, and *p*_D_ correspond to “peak” pulse intensity (*p*_I_; μmol m^−2^ s^−1^), pulse frequency (*p*_F_; i.e., 0.1–250 kHz), and pulse duration (*p*_D_; e.g., 1 μs; defined as the full width at half maximum amplitude of the pulse that is used, or FWHM), respectively (Avenson & Saathoff, 2018). In some instruments, *p*_F_ can be adjusted (Avenson & Saathoff, 2018), but *p*_I_ and *p*_D_ should, if their values are adjustable, remain constant during an experiment to ensure that the absolute intensities of the *individual* MPs remain constant. The AL and SF sources can be designed to be controlled by the same array of LEDs when they are of the same spectral quality, or color, which is ideal because different qualities of light variably penetrate the depth of a leaf (Evans, 1999; Evans et al., 2017; Nishio, 2000; Vogelmann & Evans, 2002). When they are illuminated, the AL, SF, and FR illumination sources are functionally “continuous”, namely, they are non‐modulated in nature; this distinction is meant to differentiate their intrinsic nature from that of the “pulsing” nature of the ML. The different natures of the respective light sources are about how the electronics of an instrument selectively measure modulated fluorescence (MF) associated with the MPs. The non‐modulated light sources are intended to *affect* nuanced experimental manipulations of leaf‐level photosynthesis (discussed below). At a given time during an experiment, the cumulative light that is incident on a leaf or other samples of interest is the sum of the ML, AL, SF, and FR light sources that are applied, but that cumulative intensities vary depending upon the experimental manipulation being implemented (Figure 2a).

It is critical to understand that the essence of the PAM chlorophyll *a* fluorescence technique is that while the non‐modulated light, which includes the AL, SF, and FR sources, affect various photosynthetic responses, the photons in the individual MPs of the ML source are designed to “probe,” for example, which is to say that they elicit *F*_I_'s that are reflective of, these photosynthetic processes, but without impacting them *per se*. Figure 2 (panel B) schematically illustrates the fluorescence signals associated with the ML, AL, and SF light sources. MF is depicted as being elicited in the absence of AL (darkness) *and* when both the AL and SF sources are ON, namely, the ML is ON and eliciting MF during all of these various illumination states. MF is the signal of interest and requires no a priori knowledge of *Q*_Abs_ in order to infer Φ_F_ from the detected *F*_I_'s (Equation 10; assumption #2). In contrast, continuous fluorescence (CF) is selectively elicited only when the AL and SF sources are ON. To convert CF to Φ_F_, *Q*_Abs_ must be known, as well as the spectral composition of the actinic light source. The detector of a PAM chlorophyll *a* fluorometer is protected from registering intensity changes due to stray, non‐fluorescence light by an optical filter that is solely transparent to wavelengths in the chlorophyll *a* fluorescence spectral range, which spans wavelengths from approximately 650–850 nm; various long‐pass and/or band‐pass filters can be employed to reject detection of stray light associated with the actinic and modulated light sources. Briefly, the electronic componentry of the ML source itself, and that of the detector, are designed to discriminate between the exceedingly small MF signal and the much larger CF signals (Papageorgiou, 2007; Schreiber, 1986; Schreiber, 2004). Accordingly, during the hypothetical AL illumination period (panel B), the gist of the modulation and “demodulation” electronic circuitry is that F_I_, for example, is detected just prior to (“*d*”), during the maximum amplitude of (“*e*”), and just after (“*f*”), an individual MP, respectively. Conceptually speaking, the MF signal is electronically demodulated, or discriminated, from the CF signal according to the expression:

(14) $$\mathrm{MF}=e-\left(\frac{\left(d+f\right)}{2}\right),$$

where MF is measured during all non‐modulated illumination conditions based on the same electronic principles (see points a through c and g through i, respectively). The CF signal, which is not necessarily an output of all commercially available fluorometers (Avenson & Saathoff, 2018), is depicted as being measured by detecting *F*_I_'s at points d, f, g, and i, and so on. The *critical* point is that the MF signals, the amplitudes of which can significantly vary during the dark, AL illumination, and SF illumination, are elicited by ML pulses of *constant* intensity, namely, the variable amplitudes of the MF signals during the dark, AL and SF, are not due to different ML pulse intensities *per se*; thus, they represent different processes that impact the magnitudes of Φ_F_ (Equation 10; assumption #2). Recall that Φ_F_, not F_I_ *per se*, is the “parameter” that carries information about how light energy absorbed by chlorophyll is variably partitioned. Given the constant intensity of the MPs, the consequently detected *F*_I_'s necessarily reflect “relative” Φ_F_. Thus, PAM chlorophyll *a* fluorescence has been particularly successful in providing information about various aspects of light energy conversion of both dark‐adapted and light‐adapted leaves (Juneau et al., 2005).

## 4 METHODOLOGICAL AND MATHEMATICAL DESCRIPTIONS OF KEY Φ_F_ 'S: UNDERLYING ASSUMPTIONS

### 4.1 Dark‐adapted state

**FIGURE 3:**
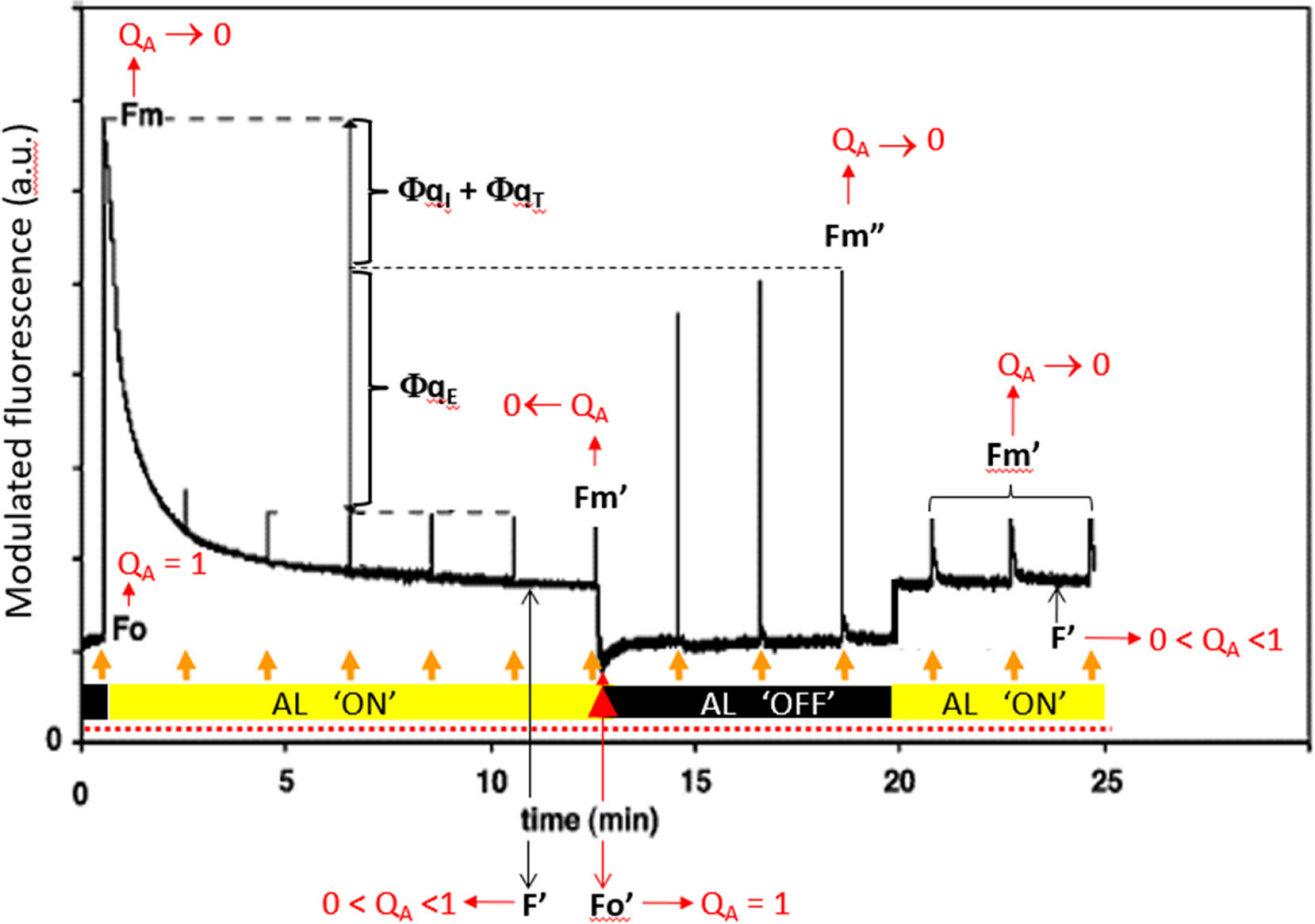
Experimental manipulation involving various non‐modulating light sources were performed to measurekey modulated fluorescence parameters. The modulated, or measuring, light (ML; dotted red line) remains “ON” throughout the entire experiment. The actinic light (AL; 750 μmol photon m^−2^ s^−1^) ‘ON’ (yellow) and ‘OFF’ (black) states are indicated by the thick bar. The red (upward‐pointing) arrow at the light‐to‐dark transition is intended to depict far‐red illumination for several seconds, illumination of which is simultaneous with cessation of AL illumination, an experimental manipulation designed to measure $F_{o}^{\prime }$. The orange arrows represent a train of brief (1 s) saturation flashes (several times full sunlight intensity). The assumed proportion of *Q*_A_ for *F*_o_, *F*_m_, *F*′, $F_{m}^{\prime }$, and $F_{m}^{\prime \prime }$ are indicated in red (see text for details and explicit equations and descriptions). The quantum yields (Φ) of *q*_E_ and (*q*_T_ + *q*_I_) are described in the text (Ahn et al., 2009), respectively. NPQ = ∑(*q*_E_ + *q*_T_ + *q*_I_). Figure was modified from the original (Müller et al., 2001) with permission

Maybe counterintuitively to new users, meaningful information about photosynthesis can be obtained from a dark‐adapted leaf (Figure 3). Illumination of a darkened leaf solely with the ML results in what is termed the “dark‐adapted, steady‐state (i.e., $\frac{d\Phi F}{\mathrm{dt}}=0$) minimum Φ_F_” (*F*_o_), which is measured in the absence of AL, SF, and FR sources, and is mathematically defined according to “lake” model assumptions as:

(15) $$\begin{array}{c} F_{o}=\Phi_{F}=\frac{k_{F}}{\sum \left(k_{F},+,k_{\mathrm{IC}},+,k_{\mathrm{ISC}},+,k_{\mathrm{PC}},*,\left(Q_{A}=1\right)\right)}. \end{array}$$

A general assumption of the PAM chlorophyll *a* fluorescence technique, when measuring *F*_*o*_, is that the *I*_I_ (Equation 13) of the ML is non‐actinic, for example, it should not be so intense as to stimulate any appreciable amount of A_Net_, nor should it cause any dynamic changes in *Q*_A_ (*Q*_A_ is assumed to equal 1). The assumption that *Q*_A_ = 1 means that the ensemble proportion of PSII reaction centers is *completely* oxidized. Therefore, it is always imperative to confirm these assumptions when using the technique with a given species and under the environmental condition(s) of interest (Fernandez‐Jaramillo et al., 2012). Additional assumptions underlying accurate determination of *F*_o_ are that all NPQ processes have completely “relaxed” or “disengaged”, as denoted by the fact that the rate constants for NPQ processes are absent in the denominator of Equation (15). An appropriately dark‐adapted leaf, or other samples of interest, is in a unique physiologic state and thus *F*_o_ and *F*_m_ (Equations 15 and 16) are best assessed after overnight dark adaptation (Adams III et al., 2006). Application of a SF (1 s in duration) to an appropriately dark‐adapted leaf is meant to elicit the ‘dark‐adapted maximum Φ_F_’ (*F*_m_):

(16) $$F_{m}=\Phi_{F}=\frac{k_{F}}{\sum \left(k_{F},+,k_{\mathrm{IC}},+,k_{\mathrm{ISC}}\right)}.$$

The first assumption concerning the accurate determination of *F*_m_ is that the *I*_I_ of the ML does not activate any NPQ; the rate constant for NPQ is absent in the denominator of Equation 16. The second assumption is that the intensity of the SF transiently causes the proportion of *Q*_A_ to approach zero, meaning *Q*_A_ → 0, and thus Equation 16 also lacks a rate constant for PSII‐mediated photochemistry. It is further assumed that the SF does not cause changes in the rate constants of the other intrinsic photophysical processes, like *k*_IC_, nor does it induce any one of a number of well‐known, additional quenching phenomena (Kramer & Crofts, 1996). This latter assumption is intrinsic to the PAM chlorophyll *a* fluorescence technique when used in conjunction with the SF method (Schreiber, 2004).

### 4.2 Light‐adapted state

Illumination of a darkened leaf by AL activates the integrated processes of photosynthesis, including proton‐coupled LEF, NPQ, and *A*_Net_ (Figure 1), the dynamics of which are consequently reflected in the following expressions of Φ_F_. Upon initial illumination, coincident with an application of the 1 s SF (Figure 3), the transient rise in Φ_F_, which reflects a *transient* decrease in *Q*_A_ due to the counteracting interplay between induction of LEF and NPQ, is followed by a progressive decay to a steady‐state condition, which could take tens of minutes to be achieved from the darkened state. The resultant Φ_F_ is termed the “light‐adapted, steady‐state minimum Φ_F_” (*F*′):

(17) $$\begin{array}{c} F^{\prime }=\Phi_{F}=\frac{k_{F}}{\sum \left(k_{F},+,k_{\mathrm{IC}},+,k_{\mathrm{ISC}},+,k_{\mathrm{PC}},*,\left[0<Q_{A}<1\right],+,k_{\mathrm{qE}},*,\left[\mathrm{Zea}\right],\left[P\right],\mathrm{\Delta pH},+,k_{\mathrm{qT}},+,k_{\mathrm{qI}}\right)}. \end{array}$$

Under steady‐state illumination, and depending on the AL intensity, the assumption (Equation 17) is that the proportion of Q_A_ will vary between 0 and 1; the magnitude of the trans‐thylakoid ΔpH and the [Zea] will also vary (Takizawa et al., 2008). The observed decrease in Φ_F_ after initial AL illumination reflects LEF and NPQ competing with fluorescence (F), as well as with IC and ISC, for dissipation of absorbed light energy. Subsequent application of a SF to a leaf that is adapted to steady‐state AL illumination results in the “light‐adapted, maximum Φ_F_” ($F_{m}^{\prime }$):

(18) $$\begin{array}{c} F_{m}^{\prime }=\Phi_{F}=\frac{k_{F}}{\sum \left(k_{F},+,k_{\mathrm{IC}},+,k_{\mathrm{ISC}},+,k_{\mathrm{qE}},*,\left[\mathrm{Zea}\right],\left[P\right],\mathrm{\Delta pH},+,k_{\mathrm{qT}},+,k_{\mathrm{qI}}\right)}. \end{array}$$

As with measurement of *F*_m_ (Equation 16), the sole assumption concerning the measurement of $F_{m}^{\prime }$ is that *Q*_A_ → 0 during the SF, as denoted by the fact that “*k*_PC*_[0 < *Q*_A_ <1]” is solely absent in Equation (18) when compared to *F′* (Equation 17). The smaller amplitude of $F_{m}^{\prime }$ in comparison to *F*_m_ (Figure 3) is indicative of the engagement of NPQ processes (∑[Φ_qE_ + Φ_qT_ + Φ_qI_]) during measurement of the former. In addition, from the steady‐state illuminated condition, turning “OFF” the AL for a short duration (2–5 s), while simultaneously turning “ON” a low intensity of FR light source, which *preferentially* excites PSI (Pfündel et al., 2013), is intended to completely oxidize the redox intermediates of PSII, as exemplified by the proportion of the singly reduced form of *Q*_A_ ($Q_{A^{-}}$) going completely oxidized (i.e., $Q_{A^{-}}$ → *Q*_A_; Pfündel et al., 2013). The resultant state of the system is characterized as the “darkened, NPQ‐engaged minimum Φ_F_” ($F_{o}^{\prime }$; Figure 3):

(19) $$\begin{array}{c} F_{o}^{\prime }=\Phi_{F}=\frac{k_{F}}{\sum \left(k_{F},+,k_{\mathrm{IC}},+,k_{\mathrm{ISC}},+,\left(k_{\mathrm{PC}}*\left[Q_{A}=1\right]\right),+,k_{\mathrm{qE}},*,\left[\mathrm{Zea}\right],\left[P\right],\mathrm{\Delta pH},+,k_{\mathrm{qT}},+,k_{\mathrm{qI}}\right)}. \end{array}$$

The assumptions of this particular experimental manipulation are that the PSII reaction centers undergo complete, yet transient, oxidation, meaning that *Q*_A_ = 1, for example, all PSII reaction centers transiently “re‐open” (Oxborough & Baker, 1997), and all components of NPQ remain as engaged as they were under steady‐state illumination. A slightly different experimental manipulation of a leaf under steady‐state illumination involves turning the AL “OFF” for a longer period of time (60–300 s), while simultaneously applying a SF, resulting in the “q_E_‐devoid, maximum Φ_F_” ($F_{m}^{\prime \prime }$; Figure 3):

(20) $$F_{m}^{\prime \prime }=\Phi_{F}=\frac{k_{F}}{\sum \left(k_{F},+,k_{\mathrm{IC}},+,k_{\mathrm{ISC}},+,k_{\mathrm{qT}},+,k_{\mathrm{qI}}\right)}.$$

These experimental manipulations are assumed to solely enable the q_E_ component of NPQ to “relax,” while simultaneously causing *Q*_A_ → 0, both changes of which are reflected in the denominator of Equation (20). After the relaxation of q_E_, abrupt re‐illumination of the leaf at the previous AL intensity will return it to the “pre‐darkened,” steady‐state illuminated condition that is characterized by the original magnitudes of *F*′ and $F_{m}^{\prime }$ (Figure 3). Such rapid fluctuations in the respective maximum Φ_F_'s ($F_{m}^{\prime }$ and $F_{m}^{\prime \prime }$) are characteristic of the significantly important, and rapidly reversible, dynamics of the q_E_ component of NPQ (Müller et al., 2001; Pfündel et al., 2013).

**FIGURE 4:**
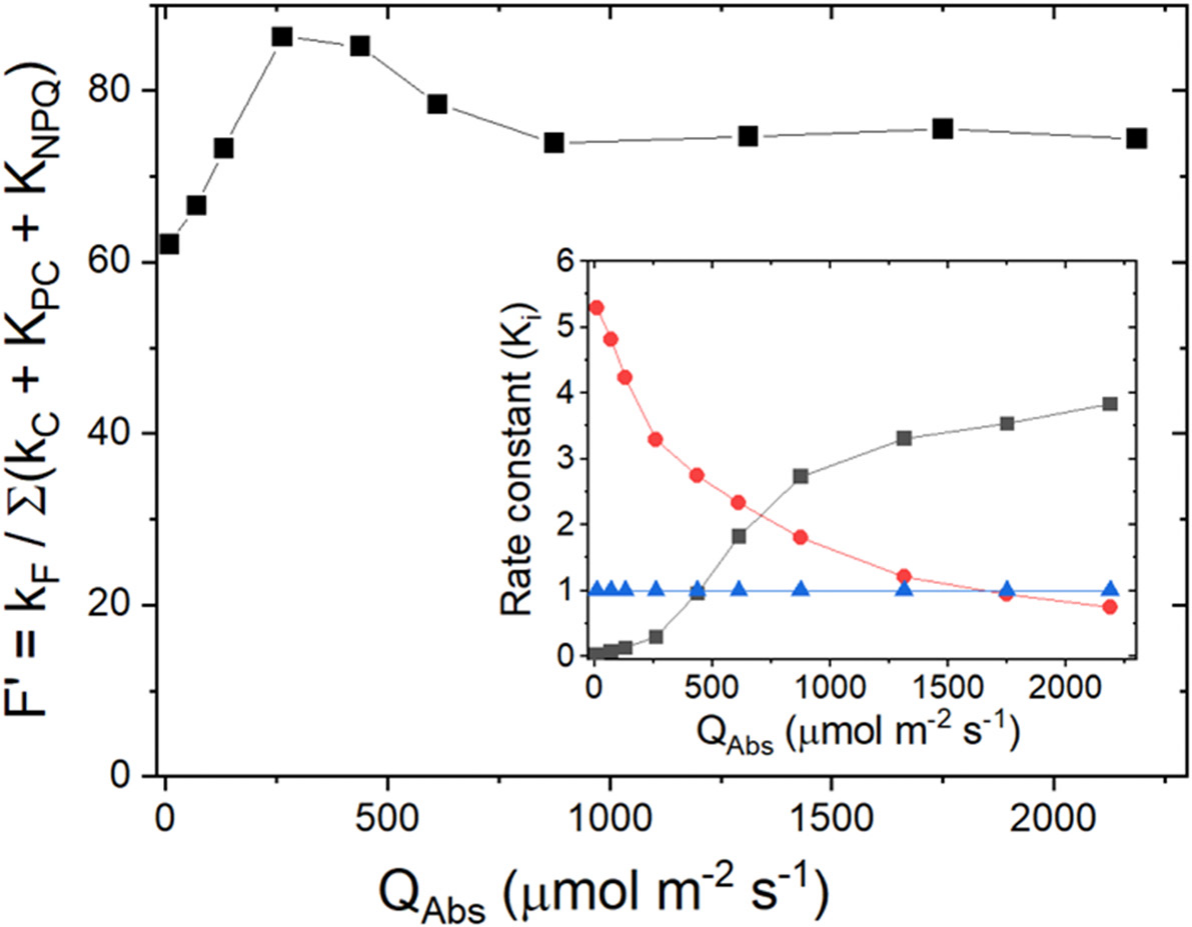
Steady‐state fluorescence yield dynamics as a function of increasing actinic light intensities A *N. tabacum* leaf was clamped into a LI‐6800 fluorometer chamber and the steady‐state, modulated fluorescence yield (Φ_F_; *F*′) was measured from 10 to 2500 μmol m^−2^ s^−1^ of the incident light, which was converted to absorbed light (*Q*_Abs_; *x*‐axis). Each step change in actinic light (AL) was followed by a 30‐min wait period to ensure steady‐state conditions. The modulated light settings were: (1) peak amplitudes of the modulated pulses = 100 μmol m^−2^ s^−1^; (2) pulse frequency = 500 Hz; and (3) pulse width = 1 μs. The chamber conditions were controlled at: (1) leaf temperature = 25°C; (2) chamber [CO_2_] = 400 μmol mol^−1^; (3) boundary layer conductance: 2.3 mol m^−2^ s^−1^; and (4) flow rate = 500 μmol air s^−1^. Inset: Normalized intrinsic and apparent rate constants (i.e., relative to *k*_C_; see below) for linear electron flux (LEF; *K*_LEF_/*k*_C_; red symbols) and non‐photochemical quenching (NPQ; *K*_NPQ_/*k*_C_; black symbols) were calculated as described in (Ahn et al., 2009; *k*_C_ = ∑[*k*_F_ + *k*_IC_ + *k*_ISC_]), where *k*_F_, *k*_IC_, and *k*_ISC_ correspond to first‐order rate constants for the decay of singlet, excited chlorophyll (^1^Chl^*^) via fluorescence (F), internal conversion (IC), and intersystem crossing (ISC) of ^1^Chl^*^ into the triplet state (^3^Chl^*^), followed by decay to the ground state. Note that *k*_C_/*k*_C_ = 1 (blue symbols) is assumed (Ahn et al., 2009)

It can be instructive to observe the relation between the dynamics of the abovementioned Φ_F_'s with the underlying pattern of changes in the relevant rate constants. Shown in Figure 4 is an example of *F′* measurements (Equation 17) as a function of increasing intensities of AL. Also, shown are the apparent rate constants (inset), *K*_PC_ and *K*_NPQ_ (Ahn et al., 2009), which exponentially decrease and sigmoidally increase, respectively, as a function of increasing AL intensity. Noticeably, *K*_PC_ initially decreases more steeply than the corresponding increases in *K*_NPQ_. However, as the AL progressively increases, *K*_PC_ begins to asymptotically approach its minimum value, whereas *K*_NPQ_ exhibits a steep rise, followed by a gradual increase toward its asymptotic maximum value. It is this disproportionate interplay between the dynamic changes in *K*_PC_ and *K*_NPQ_ that account for the transient rise of *F′*, followed by a relative settling of the signal.

## 5 METHODOLOGICAL CAVEATS: VALIDATION AND COMPENSATORY COUNTER MEASURES

### 5.1 Spectral considerations

**FIGURE 5:**
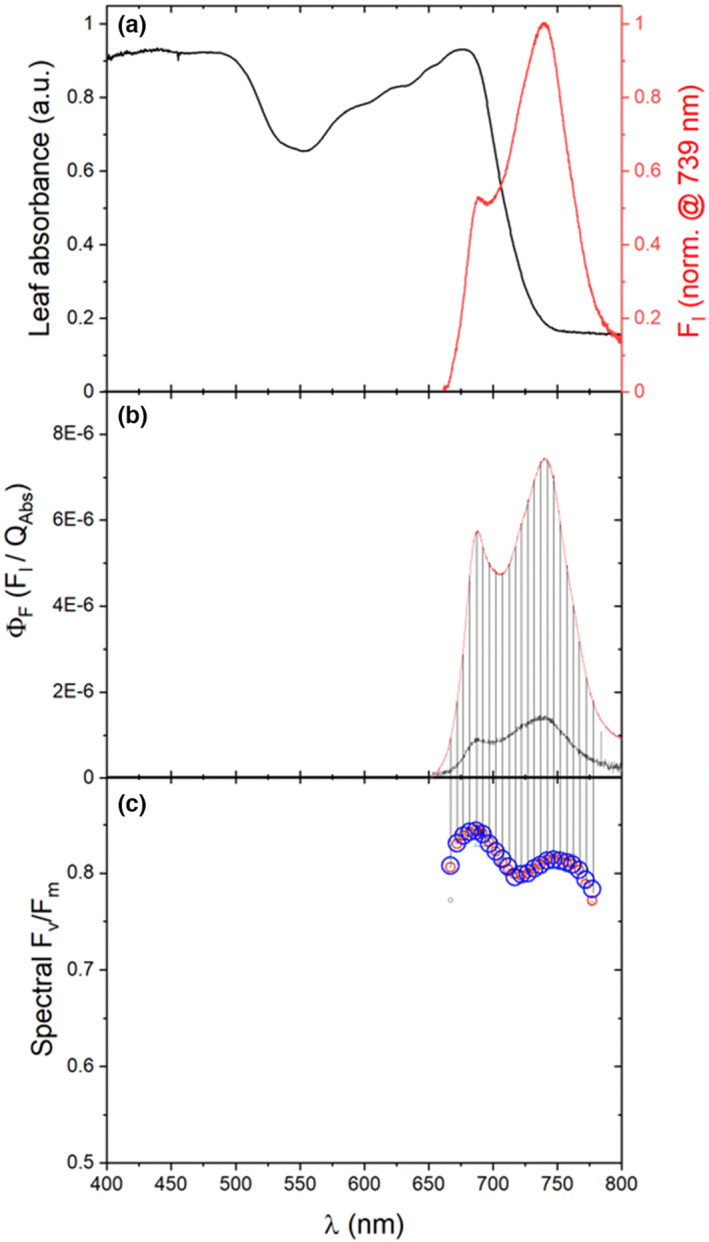
Spectroscopic features of leaf‐level phenomena (a) leaf absorbance (black line) of a *N. tabacum* leaf was measured with a LI‐1800 integrating sphere (LI‐COR Biosciences, 4647 Superior St.) coupled to a Flame® Spectrometer (Ocean Insight 3500 Quadrangle Blvd.) with 1‐nm resolution. Spectral fluorescence intensity (red line; μmol m^−2^ s^−1^) was adaxially measured from a *N. tabacum* leaf exposed to actinic light (AL) of 700 μmol m^−2^ s^−1^ in a sealed custom‐designed pulsed amplitude modulation (PAM) chlorophyll *a* fluorometer chamber of an LI‐6800 equipped with optical fibers coupled to a QEPro® Spectrometer (Ocean Insight 3500 Quadrangle Blvd.) with 0.19 nm resolution. (b) Dark‐adapted minimum (*F*_o_; black) and maximum (*F*_m_; red) spectral fluorescence intensities (μmol m^−2^ s^−1^) were measured from an *N. tabacum* leaf exposed to a low AL intensity (i.e., 10 μmol m^−2^ s^−1^) and during a rectangular flash (RF) of 5000 μmol m^−2^ s^−1^ using the above‐mentioned LI‐6800‐equipped spectrometer system. The instrument was coupled to a high‐resolution spectrometer (full instrument description in a forthcoming publication). Leaf absorbance was measured as in (A) to obtain estimates of absorbed light for the AL and RF light (*Q*_Abs_). Spectral fluorescence intensity (*F*_I_) was divided by *Q*_Abs_ to obtain spectral fluorescence yields. (c) Integration under regions (vertical lines) of the respective spectral Φ_F_s, as a function of increasing wavelength, was performed to estimate the wavelength‐dependence of *F*_v_/*F*_m_. Integration was performed over 5 nm (black symbols), 10 nm (red symbols), and 15 nm (blue symbols) increments at center wavelengths between 650 and 810 nm

An important caveat about the detection of chlorophyll *a* fluorescence from a leaf, or other samples of interest, has to do with chlorophyll absorptive and emissive interactions. Figure 5 (panel A) shows the absorption spectrum of a *Nicotiana tabacum* leaf, as well as a chlorophyll *a* fluorescence emission spectrum of a *N. tabacum* leaf under steady‐state illumination. The ratio of the ~688 to ~740 nm peaks, which correspond to what will be referred to as the “high‐energy” and “low‐energy” spectral regions, respectively, of the fluorescence emission spectrum, is ~0.528. In contrast, an in vitro experiment was previously performed by measuring the chlorophyll fluorescence spectra of chlorophyll dissolved in ethanol; the chlorophyll concentrations of the solutions were progressively increased (Gitelson et al., 1998). As a function of the initial increase in the chlorophyll concentrations of the solutions, the resultant ratio of the high‐energy‐to‐low‐energy fluorescence emission increased to a maximum of ~5, representing a 10‐fold higher ratio than that observed in the *N. tabacum* leaf; the ratio progressively decreased in solutions of higher chlorophyll concentrations. The decrease in the high‐energy‐to‐low‐energy ratio in solutions of high chlorophyll concentration was attributed to specific reabsorption of the high‐energy spectral region (Gitelson et al., 1998). The reabsorption is due to a significant overlap of the high‐energy spectral region of fluorescence emission with the longer wavelength *absorptive* spectral region of chlorophyll (Gitelson et al., 1998). Taken together, these data suggest that the significantly attenuated spectral region of chlorophyll *a* fluorescence centered at ~688 nm in *N. tabacum* (Figure 5a) is the result of significant intra‐leaf reabsorption (Franck et al., 2002; Gitelson et al., 1998). The net effect of reabsorption of the higher energy spectral region of chlorophyll *a* fluorescence in leaves is that detection of fluorescence at these wavelengths likely does not proportionally represent fluorescence from throughout the entire depth of the leaf, but rather the detected signal will be biased in fluorescence originating from the upper layers of the leaf. Fluorescence elicited within the lower portion of the leaf has a low probability of escaping the leaf and being detected (Kalaji et al., 2014). Thus, re‐absorbance of fluorescence can preclude accurate correlation between parameters that are based on fluorescence and those based on techniques that accurately reflect the entire depth of the leaf (i.e., gas exchange; Long et al., 1996). While it may seem like a solution is to measure fluorescence at the longer wavelengths, there is an alternative caveat about doing so.

The assumption about the abovementioned fluorescence parameters (Equations (15), (16), (17), (18), (19), (20)) is that they explicitly represent PSII photo‐physics. However, detection of chlorophyll *a* fluorescence within the spectral region centered at ~740 nm is problematic because both PSII and PSI emit chlorophyll *a* fluorescence at these longer wavelengths. But recall that it is only the Φ_F_ associated with PSII that provides information about how solar energy is variably partitioned into the biosphere. In leaves, PSI predominantly exhibits a single spectral region of fluorescence that is centered at ~725 nm and it undergoes little, if any, reabsorption (Franck et al., 2002). In addition, it is generally assumed that only PSII exhibits variable fluorescence, namely, changes in response to variability in AL and during SFs (Franck et al., 2002; Genty et al., 1990; Pfündel et al., 2013), although this assumption has recently been challenged (Schreiber & Klughammer, 2021), namely, whether or not PSII *alone* contributes to maximum Φ_F_'s (*F*_m_, $F_{m}^{\prime }$, and $F_{m}^{\prime \prime }$). Even if PSI does not exhibit variable fluorescence, when detecting fluorescence at longer wavelengths, the result of the constant emission of a steady‐state, minimum amount of PSI chlorophyll fluorescence will be its disproportionate contribution to the steady‐state, minimum Φ_F_ parameters (*F*_o_, *F*′, and $F_{o}^{\prime }$) in comparison to the corresponding maximum Φ_F_ parameters (*F*_m_, $F_{m}^{\prime }$, and $F_{m}^{\prime \prime }$). Since the Φ_F_ parameters are assumed to solely represent fluorescence emanating from PSII, such disproportionate contributions of PSI fluorescence will quantitatively impact estimation of key physiologic parameters that are understood to be solely associated with PSII (below; Pfündel et al., 2013).

### 5.2 Dark‐adapted state

Caveats about measuring dark‐adapted Φ_F_ parameters (*F*_o_ and *F*_m_) are associated with the relevant light sources applied. If the integrated intensity of the ML is too high, then it is possible for it to be actinic, thereby causing *Q*_A_ to be less than 1, a violation of the dark‐adapted assumptions. If the ML intensity is actinic, it means that it could even lead to engagement of LEF, NPQ, and *A*_Net_. In order to ensure that the ML is “non‐actinic,” a preliminary experiment can be performed on a darkened leaf by systematically changing the I_I_ of the ML (Equation 13) and measuring *F*_o_, *F*_m_, and/or *A*_Net_ signals (i.e., to ensure $\frac{d\Phi_{F}}{\mathrm{dt}}=0$ and $\frac{dA_{\mathrm{Net}}}{\mathrm{dt}}=0$, respectively), none of which should be characterized by any dynamics while increasing I_I_. Furthermore, given that application of a SF can potentially induce any one of a number of different auxiliary quenching mechanisms that are presumed to be absent in a darkened leaf (see Equations 15 and 16; Kramer & Crofts, 1996), effectively nullifying the accuracy of the resultant value of *F*_m_, preliminary experiments can be performed by randomly applying a series of variably intense SFs to a dark‐adapted leaf and plotting the respective values of “apparent” *F*_m_ versus SF intensity (Note: the Φ_F_ following a given SF must return to the pre‐flash level prior to applying a subsequent SF; Avenson & Saathoff, 2018). Such estimates of *F*_m_ ought to approach saturation, reflecting the fact that *Q*_A_ → 0, at a certain SF intensity and then ideally remain constant. However, decreasing values of *F*_m_ as a function of increasing SF intensities would be a clear sign that an auxiliary quenching mechanism(s) (Kramer & Crofts, 1996) was/were being induced and that lower SF intensities should be used during dark‐adapted measurements.

### 5.3 Light‐adapted state

The light‐adapted Φ_F_ parameters can be plagued by a different assortment of issues. The light‐adapted, steady minimum Φ_F_ (*F*′) can also be impacted by the *I*_I_ of the ML being too high, mostly problematic when AL intensities are low. A good rule of thumb is to examine whether the *F*_o_ signal is characterized by steady‐state under dark‐adapted conditions. If so, then the same *I*_I_ of the ML could be used for the light‐adapted state, especially at low AL intensities. It is essential to maintain a constant *p*_I_ (and *p*_D_, which is typically fixed by the manufacturer design) throughout dark‐adapted and light‐adapted measurements, otherwise, the respective parameters will not be comparable. It is also noteworthy that there is a trade‐off between the requirement of the I_I_ of the ML being non‐actinic with reasonable signal‐to‐noise (S:N) ratios. Some commercial instruments are designed to increase p_F_, consequently increasing *I*_I_, allowing for more averaging of data points per unit period during higher AL and SF intensities to improve S:N ratios when the *I*_I_ of the ML is a small proportion of total light intensity incident on the leaf (Avenson & Saathoff, 2018). A caveat about measuring $F_{m}^{\prime }$ is that it is prone to being underestimated, even by light intensities many times full sunlight (Markgraf & Berry, 1990), but several experimental methods, one of which is termed the Multiphase Flash (MPF) technique, have been implemented in several commercial systems to compensate for this potential problem (Earl & Ennahli, 2004; Loriaux et al., 2013; Markgraf & Berry, 1990).

Accurate measurement of $F_{o}^{\prime }$ is subjected to several very nuanced caveats. It is true that FR light is preferentially absorbed by PSI, an experimental manipulation that is intended, along with the AL being turned OFF, to cause *Q*_A_ to go completely oxidized (Pfündel et al., 2013). These experimental manipulations would, all things being equal, cause *F′* to decrease toward $F_{o}^{\prime }$ (Figure 3; Equations 17 and 19). However, PSII has been shown to absorb FR light (Pettai et al., 2005), which could have the effect of causing *Q*_A_ to be reduced (*Q*_A_ → $Q_{A^{-}}$), thereby precluding the necessary condition for estimation of $F_{o}^{\prime }$, namely, *Q*_A_ → 1 (Murchie & Lawson, 2013). Furthermore, the reality is that when the AL is turned off, proton flux into the thylakoid lumen via light‐induced LEF and CEF is instantaneously halted (Sacksteder et al., 2000), but the efflux of protons out of the thylakoid lumen and into the chloroplast stroma continues through the ATP synthase until the ΔpH completely collapses (Cruz et al., 2001; Figure 1). Consequently, when the AL is turned off, *q*_E_ can begin to quickly relax, an unavoidable reality that contributes to the potential difficulty of achieving an accurate estimation of $F_{o}^{\prime }$ (Equation 19).

**FIGURE 6:**
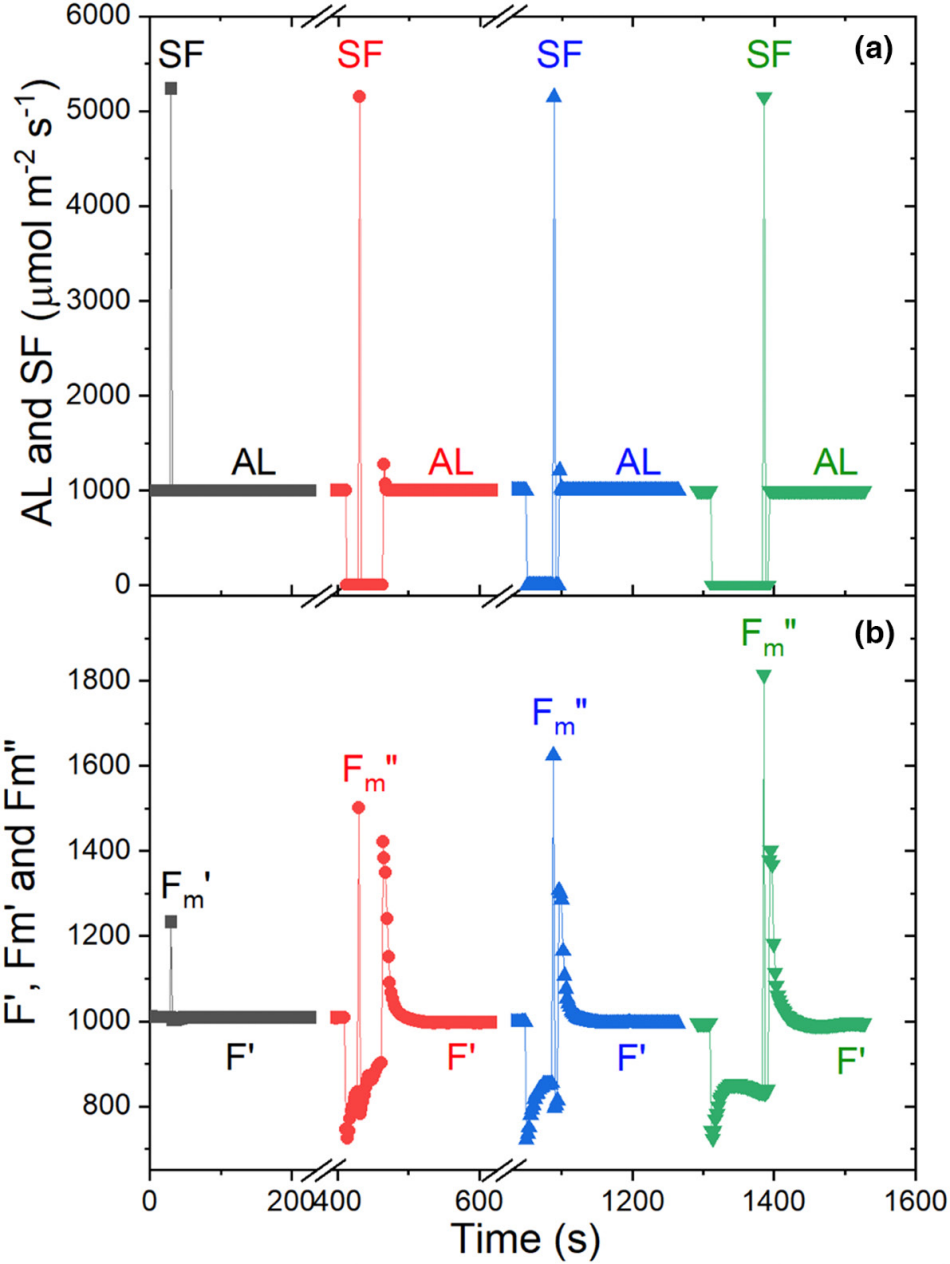
Impact of short dark periods on the estimation of $F_{m}^{\prime \prime }$. A *N. tabacum* leaf was clamped into a LI‐6800 photosynthesis instrument equipped with a PAM chlorophyll *a* fluorometer chamber (LI‐COR biosciences, 4647 Superior St.). The leaf was exposed to an actinic light (AL) intensity of 1000 μmol m^−2^ s^−1^ long enough to reach steady‐state Φ_F_ (*F*′), after which (a) 5000 μmol m^−2^ s^−1^ saturation flashes (SFs) were applied to measure $F_{m}^{\prime }$. The AL was firstly turned OFF for 18 s and another SF was applied to measure $F_{m}^{\prime \prime }$. The leaf was then re‐exposed to the AL to reestablish *F*′, following which the AL was turned OFF again and another SF was applied after 38 s. This was repeated one more time; however, the post actinic light‐dark period was 78 s before applying the SF. (b) Thus, the impact of short (tens of seconds) dark periods, which allow energy‐dependent quenching (*q*_E_) to relax, on the estimation of $F_{m}^{\prime \prime }$ was measured

The tendency for *q*_E_ to quickly relax can be experimentally demonstrated. Illuminated minimum and maximum Φ_F_ parameters, namely, *F′* and $F_{m}^{\prime }$, as well as a series of estimates of $F_{m}^{\prime \prime }$, are shown in Figure 6. Relative to $F_{m}^{\prime }$, the series of estimates of $F_{m}^{\prime \prime }$ progressively increased as a function of longer periods of time during light‐to‐dark transitions, suggesting that q_E_ rapidly relaxes during short dark periods (Equation 18 compared to Equation 20). Therefore, in conjunction with the possible effects of PSII absorption of FR light, and consequent reduction of *Q*_A_ → $Q_{A^{-}}$, the rapid relaxation of *q*_E_ renders measurement of $F_{o}^{\prime }$ prone to ambiguity. Therefore, derived estimates of $F_{o}^{\prime }$ that are based on parameters measured with much less ambiguity may be preferred and can be estimated as (Oxborough & Baker, 1997):

(21) $$F_{o}^{\prime }=\frac{F_{o}}{\frac{F_{v}}{F_{m}}+\frac{F_{o}}{F_{m}^{\prime }}}.$$

Estimates of $F_{m}^{\prime \prime }$, presumably reflecting *complete* and *specific* relaxation of q_E_, can potentially reflect overlap of relaxation of both *q*_E_ and *q*_T_ (Equation 20), a circumstance that would cause uncertainties in the estimation of $F_{m}^{\prime \prime }$, and consequently *q*_E_. There are independent phenomena that reflect q_E_, an example of which are changes in absorbance at 535 nm (Δ*A*_535_; Li et al., 2004; Li et al., 2002). Therefore, comparison of estimates of q_E_ based on values of “apparent” $F_{m}^{\prime \prime }$ over time following cessation of AL illumination with simultaneous measurements of Δ*A*_535_ is a means of verifying the appropriate duration of the dark period for accurate determination of $F_{m}^{\prime \prime }$ and *q*_E_ (Figures 3 and 6).

## 6 FLUORESCENCE‐DERIVED PHYSIOLOGICAL PARAMETERS AND THEIR UTILITY

Given the current and intense interest in more explicitly understanding SIF and in improving Y_P_ via genetically modulating NPQ dynamics (below), there is somewhat of a renewed interest in estimating fluorescence‐derived, physiologic parameters, which provide useful information about leaf‐level photosynthesis. These parameters could also be direct indicators strongly associated with a plant's energy conversion efficiency and stress responses.

### 6.1 Dark‐adapted state

PAM chlorophyll *a* fluorescence parameters measured on dark‐adapted leaves can provide information about how optimally key aspects of the energy conversion “machinery” will operate when leaves are subsequently illuminated (Adams III et al., 2006). Through a series of algebraic manipulations, the “maximum quantum yield of PSII‐mediated electron transfer” (*F*_v_/*F*_m_) can be expressed as:

(22) $$\begin{array}{c} \frac{F_{v}}{F_{m}}=\frac{F_{m}-F_{o}}{F_{m}}=\frac{k_{\mathrm{PC}}\left(Q_{A}=1\right)}{\sum \left(k_{F},+,k_{\mathrm{IC}},+,k_{\mathrm{ISC}},+,k_{\mathrm{PC}},\left(Q_{A}=1\right)\right)}, \end{array}$$

where *F*_*v*_ */F*_*m*_ is especially informative when measured after overnight dark adaptation because explicit assumptions can then be made about the underlying phenomena, thereby allowing information to be unambiguously inferred about relevant physiologic processes (Adams III et al., 2006; Pearcy et al., 1985). In healthy leaves under environmentally benign conditions or non‐environmentally stressed conditions, such an extended period of darkness allows the ΔpH component of *pmf* to completely collapse, thereby enabling the [Zea] to be fully converted to violaxanthin, the non‐quenching form of the pigment (Niyogi, 1999). Note that in dark‐adapted, over‐wintering trees, NPQ can remain engaged overnight (Adams III et al., 2006). In addition, mobile light‐harvesting complexes that underwent intra‐complex exchange between PSII and PSI in the light, referred to above as state‐transitions, or *q*_T_, will have had time to revert to their respective dark‐adapted associations. The upshot is that all components of NPQ, namely, *q*_E_, *q*_T_, and *q*_I_, will have had time to completely recover, conditions that are essential for accurate determination of *F*_v_/*F*_m_ (Adams III et al., 2006). The assumption about complete disengagement of these processes is reflected in the fact that the denominator of Equation (22) lacks rate constants for all components of NPQ. In addition, the ensemble of PSII complexes is assumed to be completely oxidized after such prolonged dark adaptation, an assumption that is also reflected in the fact that *Q*_A_ = 1 in the denominator of Equation (22). It is noteworthy that an improper *I*_I_ of the ML source (i.e., it is too intense) can cause erroneous estimation of *F*_v_ */F*_m_ due to the combined effect of changes in *F*_o_ and *F*_m_. In healthy leaves that have been sufficiently dark‐adapted, values of *F*_v_ */F*_m_ are highly conserved, with typical values having been previously shown to average ~ 0.83 over a wide range, and number (*n* = 44), of species (Björkman & Demmig, 1987). Therefore, assuming the I_I_ of the ML does not itself cause *F*_v_ */F*_m_ to be underestimated, estimates of *F*_v_ */F*_m_ are an indication of the relative health of a plant.

Different stress‐induced patterns of *F*_o_ and *F*_m_ can cause a lowering of *F*_v_ */F*_m_. From a mathematical perspective (Equations 15 and 16), stress‐induced decreases in *F*_v_ */F*_m_ can be indicative of an increase in *F*_o_, a decrease in *F*_m_, and/or a combination of a simultaneous increase in *F*_o_ and a decrease in *F*_m_ (Tyystjärvi & Aro, 1996). According to the expression for *F*_o_, it could be overestimated under environmental stress conditions due to either *Q*_A_ ≠ 1 or decreases in *k*_IC_ and/or *k*_ISC_. It is possible to distinguish between these two possibilities. On the one hand, the “potential activity” of PSII (*F*_v_ */F*_o_) can be defined as (Wu & Bao, 2011):

(23) $$\frac{F_{m}-F_{o}}{F_{o}}=\frac{k_{\mathrm{PC}}\left(Q_{A}=1\right)}{\sum \left(k_{F},+,k_{\mathrm{IC}},+,k_{\mathrm{ISC}}\right)}.$$

On the other hand, “photoinactivation” (*P*_I_) of PSII can be expressed as (Dominy & Baker, 1980):

(24) $$P_{I}=\frac{1}{F_{o}}-\frac{1}{F_{m}}=\frac{k_{\mathrm{PC}}\left(Q_{A}=1\right)}{k_{F}}.$$

In comparison to unstressed conditions, simultaneous decreases in *F*_v_ */F*_o_ and *P*_I_ would be indicative of “*k*_PC*_(*Q*_A_ ≠ 1)” (Equations 15 and 16), a possible indication of “functional” disconnection of the light‐harvesting antennae from the reaction centers of PSII (Adams III et al., 2006; Tyystjärvi & Aro, 1996). However, an increase in *F*_v_ */F*_o_ with no change in *P*_I_ would be indicative of decreases in *k*_IC_ and *k*_ISC_. Functional disconnection of the antenna from the reaction centers could be due to either light‐harvesting complexes physically disassociating from reaction centers (Björkman & Demmig‐Adams, 1995) or damage to the reaction centers themselves (Melis, 1999; Tyystjärvi & Aro, 1996). Both changes would cause *F*_o_ to increase; the former would do so because energy absorbed in the antennae could not be transferred to the functional reaction centers (Oxborough, 2004). The effect of this would be an increase in the probability of the absorbed energy being dissipated as fluorescence in the antennae. In the latter situation, even though the antennae are physically connected to the reaction centers, energy transfer could not “functionally” occur to the nonfunctional reaction centers and thus the probability of the energy being dissipated as fluorescence in the antennae would increase. These changes would be indicative of environmental stress. Sustained NPQ in dark‐adapted leaves can also be indicative of environmental stress (Melis, 1999). Recall that neither equations for *F*_o_ nor *F*_m_ contain a rate constant for NPQ, which is an assumption. Sustained NPQ in dark‐adapted leaves, which has been previously reviewed (Adams III et al., 2006; Melis, 1999), predicts that *F*_o_ and *F*_m_ will be disproportionately diminished. These NPQ changes will consequently cause a decrease in estimates of *F*_v_/*F*_m_.

While *F*_v_/*F*_m_ is a popular parameter for evaluating plant “stress” (Jägerbrand & Kudo, 2016; Lotfi et al., 2020; Sharma et al., 2015; Sharma et al., 2017), diminished values can often be due to the combined effects of high light and other stress factors in the field (Grieco et al., 2020). Therefore, its use as an explicit stress detector should be approached with caution. For example, *F*_v_/*F*_m_ cannot monitor the early effects of drought stress in the field, until and unless a specific level of dehydration is reached (Burke, 2007; Kalaji et al., 2017). In addition, Yordanov et al. (2001) pointed out that *F*_v_/*F*_o_ was more sensitive to drought stress than *F*_v_/*F*_m_, which can usually detect severe heat stress, for example, above 45°C stress events in cotton (Crafts‐Brandner & Law, 2000). However, *F*_v_/*F*_m_ also showed a good sensitivity when it was used in the detached tomato leaf discs (Willits & Peet, 2001).

Contributions of PSI fluorescence (above) can artificially cause an apparent underestimation of *F*_v_/*F*_m_. For example, shown in Figure 5b are “Φ_F_ emission spectra” measured under steady‐state, dark‐adapted conditions and during a subsequent SF; thus, the spectra consequently represent dark‐adapted minimum and maximum “spectral Φ_F_.” Integration of a progressive series of distinct regions under the respective spectra was used to obtain a series of values of spectral *F*_o_ and *F*_m_ from which estimates of *F*_v_/*F*_m_ as a function of increasing wavelength were obtained (Figure 5C). The spectrally‐derived values of *F*_v_/*F*_m_ exhibited maxima of ~0.84 and 0.81 at ~688 and ~750 nm, respectively. The tendency for the values of *F*_v_/*F*_m_ to progressively decrease as a function of increasing wavelength is fully consistent with PSI fluorescence emission disproportionately, and increasingly, contributing to the integrated values of *F*_o_ and *F*_m_ across the longer‐wavelength spectral regions. PSI has been measured to contribute 30% and 50% to *F*_o_ within the longer wavelength region in C_3_ and C_4_ species, respectively (Genty et al., 1990).

### 6.2 Light‐adapted state

Φ_PSII_ is a commonly reported fluorescence parameter and can be derived as:

(25) $$\begin{array}{c} \Phi_{\text{PSII}}=\left[\frac{\left(F_{m}^{\prime }-F^{\prime }\right)}{\left(F_{m}^{\prime }-F_{o}^{\prime }\right)}*\frac{F_{o}^{\prime }}{F^{\prime }}\right]\left[\frac{\left(F_{m}^{\prime }-F_{o}^{\prime }\right)}{F_{m}^{\prime }}\right]=\\ Q_{A}*\frac{k_{\mathrm{PC}}}{\sum \left(k_{F},+,k_{\mathrm{IC}},+,k_{\mathrm{ISC}},+,k_{\mathrm{PC}},+,k_{\mathrm{qE}}\right.\left[\mathrm{Zea}\right]\left[P\right]\mathrm{\Delta pH}+\left.k_{\mathrm{qT}},+,k_{\mathrm{qI}}\right)}. \end{array}$$

This equation independently expresses the product of the proportion of oxidized *Q*_A_, expressed according to Kramer, Johnson, et al. (2004), and the “maximum, light‐adapted quantum yield of PSII electron transfer,” respectively (Baker et al., 2007). Expression of Φ_PSII_ in this way conveys the notion that its value is ultimately the product of two independently changing variables in response to light and environmental stress. When expressed as above, the intrinsic rate constant for PSII electron transfer (*k*_PC_) is assumed to remain constant and behaves independently of *Q*_A_. As such, the dynamics of the maximum, light‐adapted quantum yield of PSII will reflect changes in the composite processes of NPQ, whereas the dynamics of *Q*_A_ will reflect the ability of the reaction centers to mediate electron transfer, potentially reflecting that reaction centers could be damaged (Baker et al., 2007). Thus, distinct details about regulated light capture versus damage to reaction centers can be discerned by independently assessing these various components. Regulation of light capture by *q*_E_ and *q*_T_ is reversible, namely, on the seconds to tens‐of‐minutes timescale and does not require energy expenditure *per se*, whereas damage to reaction centers (*q*_I_), while slowly reversible, requires energy expenditure (Melis, 1999; Tyystjärvi & Aro, 1996). In comparison to *F*_v_/*F*_m_ (Equation 22), measurements of Φ_PSII_ in the field have an advantage in early detection of nitrogen stress (Cheng et al., 2001), water stress (Kalaji et al., 2017), and heat stress (Sinsawat et al., 2004). However, *F*_v_/*F*_m_ may only need to be corrected for PSI fluorescence, while Φ_PSII_ may need correction for both PSI fluorescence and possibly for underestimation of $F_{m}^{\prime }$ (see above). Whether or not such early stress detection can be increasingly taken advantage of needs to be further explored.

Φ_PSII_ is particularly important as a means of quantifying LEF, which itself can contribute to highly meaningful information (Maxwell & Johnson, 2000), and it can be quantified as:

(26) $$\mathrm{LEF}=\Phi_{\text{PSII}}*\alpha *\beta *Q_{\mathrm{Inc}},$$

where *α*, *β*, and *Q*_Inc_ correspond to leaf absorbance, the fraction of photons partitioned to PSII versus PSI, and the incident photosynthetically‐active radiation (PAR), respectively. In order to obtain estimates of LEF that are quantitatively comparable to parameters based on, for example, gas‐exchange, it is critical to know α, which can significantly vary from 0.7 to 0.9 as demonstrated by Eichelmann et al. (2004). Species type, environmental stress, and light‐dependent chloroplast movements can potentially affect the value of *α* (Carter & Knapp, 2001; Davis et al., 2011; Osborne & Raven, 1986). In the literature, a default value of 0.84 is often assumed and used for *α* (Pan et al., 2018), as is equal partitioning of photons to both PSI and PSII (i.e., *β* is often assumed to have a value of 0.5; Murchie & Lawson, 2013). It should be emphasized, particularly because accurate comparisons between LEF and parameters based on other techniques are often desired, that these default values may not always hold true, especially considering that accumulation of non‐photosynthetic pigments and environmental stress factors can potentially change both *α* and *β* (Baker, 2008). One reason that knowledge of *α* is critical is that not all *Q*_Inc_ impinging on a leaf is ultimately absorbed and *α* enables the absorbed amount of light (*Q*_Abs_) to be measured (Long et al., 1996). Explicit estimation of α is non‐trivial since it requires a “white” light source, an integrating sphere coupled to a spectrometer, and knowledge of the spectrum of the light source used during experiments (Gitelson et al., 2003). Alternatively, a simple function between chlorophyll content (μmol m^−2^) and *α* can be established based on the assumption that chlorophyll is the predominant pigment by which light is absorbed (Evans & Poorter, 2001):

(27) $$\alpha =\frac{\left[\mathrm{Chl}\right]}{\left[\mathrm{Chl}\right]+76}.$$

In addition, Yin et al. (2009) proposed a method to estimate the product of *α* and *β* (*αβ*) under low (2%) O_2_. Under such conditions, the linear slope of *A*_net_ plotted against (*Q*_Abs_*Φ_PSII_)/4 is assumed to represent the product of α and β, while the intercept is assumed to represent daytime respiration (*R*_L_; Pons et al., 2009). In the context of measuring chlorophyll *a* fluorescence, accurate estimation of *αβ* itself is predicated on accurate estimation of Φ_PSII_, which, as mentioned above, is fraught with various caveats.

Given that Φ_PSII_ can be used to quantify LEF, a useful link between fluorescence‐derived parameters and those based on the gas exchange can be made. Gross CO_2_ assimilation (*A*_G_) can be derived from measurements of gas exchange as:

(28) $$A_{G}=A_{\mathrm{Net}}–R_{d},$$

where *R*_d_ represents respiration and is often approximated as the value of *A*_Net_ that is measured in complete darkness (Atkin et al., 1997). The relationship between LEF and *A*_G_ provides a measurement of the electron requirement of CO_2_ assimilation and is commonly obtained as the slope of a liner fit of LEF vs. *A*_G_ (Earl & Ennahli, 2004; Loriaux et al., 2013). The electron requirement of CO_2_ assimilation is, for example, indicative of the type of photosynthesis (i.e., C_3_ vs. C_4_) that a particular plant species employs (Oberhuber et al., 1993). Note that photorespiration significantly occurs in C_3_ species (Busch, 2020), a process that consumes ATP and NADPH over and above that which is used during *A*_Net_, whereas C_4_ species exploit CO_2_ concentrating mechanisms to significantly minimize photorespiration (Oberhuber et al., 1993). Thus, the electron requirements for the two types of species are quite different, which can be useful for discerning between ecologically distinct species in nature. By combining estimates of LEF with the “RUBP‐regeneration‐limited” equation for *A*_Net_ (Long & Bernacchi, 2003), an expression for *g*_m_ can be derived (Harley et al., 1992) based on what has been termed the “variable J” method (Harley et al., 1992; Note: fluorescence‐derived LEF is substituted for ‘J’ in the following):

(29) $$g_{m}=\frac{A_{\mathrm{Net}}}{C_{i}-\frac{\Gamma^{*}\left[\mathrm{LEF}+8\left(A_{\mathrm{Net}}+R_{d}\right)\right]}{\left[\mathrm{LEF}-4\left(A_{\mathrm{net}}+R_{d}\right)\right]}}.$$

Traditionally, estimates of LEF have not been used in this expression, but rather its use in the equation became more common with the development of instruments capable of simultaneously measuring both gas exchange and PAM chlorophyll *a* fluorescence (Flexas et al., 2008; Harley et al., 1992). The underlying bases of changes in *g*_m_ have been under intense investigation (Flexas et al., 2012), especially in the context of understanding drought tolerance (Flexas et al., 2012). Given the importance of such research, it is relevant to point out that estimates of g_m_ by Equation 29 are particularly sensitive to uncertainties in estimates of LEF (Harley et al., 1992). One potential source of uncertainty in estimating LEF involves the potential for underestimation of $F_{m}^{\prime }$ due to non‐saturating SF intensities (Equation 18), so in the context of measuring *g*_m_, the abovementioned MPF method may be necessary to compensate for this uncertainty (Loriaux et al., 2013). Estimates of *g*_m_ have been shown to vary at different CO_2_ concentrations (Xiong et al., 2015), which can occur in the chloroplast under environmental stress. Moualeu‐Ngangue et al. (2017) proposed a new method to estimate photosynthetic parameters without taking a value of *g*_m_ through a linear regression between an *A*–*C*_i_ curve and an *A*–*C*_c_ curve. However, potential errors may occur in this method if the uncertainties in $F_{m}^{\prime }$ and PSI fluorescence are ignored.

Estimates of LEF can also provide nuanced information by comparing it to parameters based on DIRK analyses of the ECS. In addition to estimates of $g_{H}^{+}$ and the relative magnitude of the light‐induced *pmf*, DIRK analyses of the ECS can also provide estimates of the flux of protons ($\upsilon_{H}^{+}$) into the thylakoid lumen via the *combination* of both LEF and CEF (Avenson et al., 2005; Cruz et al., 2005; Livingston et al., 2010):

(30) $$\upsilon_{H}^{+}=\gamma *\mathrm{LEF}+\delta *\mathrm{CEF},$$

where *γ* and *δ* represent the proton‐to‐electron ratios (H^+^:e^−^) of LEF and CEF, respectively (Cruz et al., 2005). Thus comparisons between LEF (Equation 26) and $\upsilon_{H}^{+}$ can provide a relative assessment of the contribution of CEF to the energy budget of photosynthesis (Walker et al., 2014; Walker et al., 2020). CEF has been proposed to have various roles, including protection of the photosynthetic apparatus via modulation of q_E_ (Avenson et al., 2004; Avenson et al., 2005; Kanazawa & Kramer, 2002), thereby minimizing the production of ROS (Foyer et al., 2012). Since CEF acidifies the thylakoid lumen over and above that which is associated with LEF, it has also been proposed to play a role in balancing the output ratio of ATP:NADPH with fluctuating metabolic demands (Kramer et al., 2003; Livingston et al., 2010).

Given the many fluorescence‐derived parameters that can be measured, it is often useful to make intra‐comparisons to gain novel insights. Comparison of q_E_ versus LEF, for example, can provide unique information about the dynamics of leaf‐level photosynthesis in response to environmental stress. Although q_E_ can be estimated by several different mathematical conventions (Ahn et al., 2008; Kramer, Johnson, et al., 2004; Maxwell & Johnson, 2000), the quantum yield of *q*_E_ (Φ_qE_), which implements the same mathematical formalism as Φ_PSII_ (Equation 25), can be estimated as (Ahn et al., 2009):

(31) $$\begin{array}{c} \Phi_{\mathrm{qE}}=\frac{F_{m}^{\prime \prime }-F_{m}^{\prime }}{F_{m}^{\prime \prime }}*\frac{F^{\prime }}{F_{m}^{\prime }}\\ =\frac{k_{\mathrm{qE}}\left[\mathrm{Zea}\right]\left[P\right]\mathrm{\Delta pH}}{\sum \left(k_{F},+,k_{\mathrm{IC}},+,k_{\mathrm{ISC}},+,k_{\mathrm{PC}},\left(0<Q_{A}<1\right),+,k_{\mathrm{qE}},\left[\mathrm{Zea}\right],\left[P\right],\mathrm{\Delta pH},+,k_{\mathrm{qT}},+,k_{\mathrm{qI}}\right)}, \end{array}$$

*q*_E_ and LEF were previously measured over a wide range of light intensities and different concentrations of CO_2_ and O_2_, following the protocol of Kanazawa and Kramer (2002), to mimic an increase in biochemical limitation, as occurs during the combination of drought and changes in light intensities (Lal & Edwards, 1996). Such biochemical limitation is known to lower *A*_Net_ and LEF. Estimates of q_E_ over a large range of light intensities and the three different combinations of CO_2_ and O_2_ concentrations exhibited a series of three discontinuous relationships versus concomitant estimates of LEF (Avenson et al., 2004). In effect, the data were consistent with q_E_ becoming more “sensitive” to LEF (Kanazawa & Kramer, 2002), as indicated by the fact that lower fluxes of LEF under biochemical limitation, conditions that were experimentally brought about by lowering the CO_2_ concentration, generated the same amount of q_E_ as during control conditions, during the latter of which LEF was actually higher. This phenomenon has come to be termed “q_E_ sensitivity” and is a response for plant survival during drought (Avenson et al., 2005; Kanazawa & Kramer, 2002; Virlouvet et al., 2018). It was hypothesized that increased q_E_ sensitivity could be due to (Avenson et al., 2004): (1) increased CEF; (2) decreased $g_{H}^{+}$; (3) an increased proportion of the *pmf* being stored as ΔpH; (4) an effective increase in the ΔpH‐dependent responsiveness of the PSII‐intrinsic mechanism of *q*_E_. It was found that the majority of the resultant increases in q_E_ sensitivity could be attributed to diminished g_H_^+^, consistent with previous findings (Kanazawa & Kramer, 2002), although more *pmf* was also partitioned into ΔpH under the most severe stress conditions (Avenson et al., 2004). Interestingly, Kohzuma et al. (2009) found that a combination of decreased $g_{H}^{+}$ and increased CEF could account for the observed increases in q_E_ sensitivity during severe drought stress under field conditions.

NPQ is receiving significant interest in the context of improving yield (Zhu et al., 2010) and it can be estimated from Φ_F_ parameters (Equations 16 and 18):

(32) $$\mathrm{NPQ}=\frac{\left(F_{m}-F_{m}^{\prime }\right)}{F_{m}^{\prime }}=\frac{k_{\mathrm{NPQ}}}{\sum \left(k_{F}+k_{\mathrm{IC}}+k_{\mathrm{ISC}}\right)}.$$

Dynamic leaf‐level processes, as measured by various chlorophyll *a* fluorescence parameters, have been gaining particular interest in recent years (McAusland et al., 2019; Zhang et al., 2019), with one of the primary objectives being to monitor the reversibility of NPQ dynamics that occurs during dark‐to‐light and light‐to‐dark transitions (Zhang et al., 2019). The reversible kinetics of NPQ were previously hypothesized to be capable of being “enhanced” as a means of specifically improving *ε*_c_ (Zhu et al., 2010). Kromdijk et al. (2016) reported that genetically‐enhanced acceleration of the rapidly reversible kinetics of NPQ (Niyogi et al., 2005) correlated with enhanced *A*_Net_ during fluctuations in light intensity, circumstances that are intermittently but routinely experienced by leaves in nature (Külheim et al., 2002). Furthermore, under field conditions, the transgenic plants accumulated more root, shoot, and stem biomass than the control plants (Kromdijk et al., 2016). Similarly, Hubbart et al. (2018) demonstrated that enhanced NPQ capacity, which was achieved by genetically overexpressing PsbS, reduced the onset of photoinhibition and thereafter improved rice biomass accumulation and yield under variable light intensities. The combined results support the hypothesis that enhanced NPQ reversibility can indeed increase ε_c_ (Zhu et al., 2010).

Assessment of the redox state of PSII itself can be particularly useful in field studies. Q_A_ is a measurable representation of the PSII redox state and can be estimated as (Kramer, Johnson, et al., 2004):

(33) $$Q_{A}=q_{L}=\frac{F_{m}^{\prime }-F^{\prime }}{F_{m}^{\prime }-F_{o}^{\prime }}*\frac{F_{o}^{\prime }}{F^{\prime }}.$$

During dynamic changes in light intensities, the rates at which A_Net_ and g_s_ reach steady‐state can be uncoupled, giving rise to a potential for diminished water use efficiency. For example, delayed stomatal closure in response to decreased light was shown to unnecessarily increase water loss (McAusland et al., 2016). Kromdijk et al. (2019) modified a model describing the kinetics of *g*_s_ by additionally incorporating *Q*_A,_ the redox potential of which has been extensively studied (Krieger et al., 1995), enabling *g*_s_ to be monitored using a readily measurable fluorescence parameter (1 − *q*_L_). Taken together, both NPQ dynamics, a commonly measured fluorescence parameter (Müller et al., 2001), and gas exchange strongly suggest that PAM chlorophyll *a* fluorescence parameters can be used for assessing critical determinants of *Y*_P_ improvement, namely, detailed aspects of the coordinated processes of leaf‐level photosynthesis.

## 7 CONCLUDING REMARKS

Global demand for food is increasing, thereby requiring without any delay, enhanced agricultural productivity. Importantly, the informative function of chlorophyll *a* fluorescence measurement has been increasingly shown to have consequential impacts on crop improvement programs. Chlorophyll *a* fluorescence provides comprehensive information about plant light use efficiency and by extension, crop growth. The selection of stress‐resistant genotypes is a particularly useful application of chlorophyll *a* fluorescence measurement. This work is meant as an update to previous chlorophyll fluorescence reviews, and some extent, as an introduction for new users of fundamental instrumentation issues. Finally, it was emphasized herein that an accurate measurement for chlorophyll *a* fluorescence is crucial for testing specific photosynthetic responses. We introduced and emphasized the importance, the principles, the “pit‐falls,” and the basic equations of chlorophyll *a* fluorescence analysis.

## CONFLICT OF INTEREST

The authors declare no conflict of interest.

## Data Availability

The data that support the findings of this study are openly available in figshare at https://doi.org/10.6084/m9.figshare.19214148
